# Effect of High Temperature and Drought on the Fatty Acid Composition of Oil of Different Linseed Varieties and Its Association with Gene Expression

**DOI:** 10.3390/ijms27188006

**Published:** 2026-09-09

**Authors:** Gleb N. Vladimirov, Sergey V. Osipenko, Liubov V. Povkhova, Anton A. Bashilov, Yury I. Kostyukevich, Eugene N. Nikolaev, Tatiana A. Rozhmina, Aleksey A. Gryzunov, Elizaveta A. Ivankina, Ekaterina M. Dvorianinova, Arthur G. Yablokov, Elena V. Borkhert, Alexander A. Arkhipov, Elena N. Pushkova, Alexey A. Dmitriev, Nataliya V. Melnikova

**Affiliations:** 1 The Center for Bio- and Medical Technologies, 121205 Moscow, Russia; gleb.vladimirov@gmail.com (G.N.V.); ossipenko9191@gmail.com (S.V.O.); anton_bashilov@mail.ru (A.A.B.); yura542@gmail.com (Y.I.K.); ennikolaev@gmail.com (E.N.N.); 2 Engelhardt Institute of Molecular Biology, Russian Academy of Sciences, 119991 Moscow, Russia; povkhova@yandex.ru (L.V.P.); sigova1567@gmail.com (E.A.I.); eugenevarenyuk@gmail.com (E.M.D.); melarsoprol@mail.ru (A.G.Y.); sashai@inbox.ru (E.V.B.); arkhipov.aleksandr2.0@gmail.com (A.A.A.); pushkova18@gmail.com (E.N.P.); alex_245@mail.ru (A.A.D.); 3 Federal Research Center for Bast Fiber Crops, 172002 Torzhok, Russia; tatyana_rozhmina@mail.ru; 4 All-Russian Scientific Research Institute of Refrigeration Industry—Branch of V.M. Gorbatov Federal Research Center for Food Systems of Russian Academy of Sciences, 127422 Moscow, Russia; grizu-nov@rambler.ru

**Keywords:** flax, fatty acids, linseed oil, temperature, drought, mass spectrometry, gene expression

## Abstract

Flax is an important food and industrial crop. Linolenic acid (LIN) content of linseed oil differs greatly among varieties, ranging from 2% to 70%. In addition to genetic factors, environmental conditions can also significantly impact the fatty acid composition of linseed oil. Our study aimed to compare the effects of different temperature and watering conditions on the fatty acid composition of oil from nine flax varieties with diverse LIN content induced by genotype. Elevated temperature with reduced watering resulted in increased oleic acid (OLE) levels in all varieties. Decreases in linoleic acid (LIO) and LIN were also observed, though these trends were less pronounced in genotypes with low LIN content. Using our previously obtained transcriptomic data for seeds of the same flax varieties grown under the same conditions, we found that elevated temperature with reduced watering caused the maximum expression levels to shift to earlier development stages and shortened the period of high expression for the key *fatty acid desaturase* (*FAD*) genes: *FAD2a-1*, *FAD2a-2*, and *FAD2b-2* (OLE to LIO desaturation) as well as *FAD3a* and *FAD3b* (LIO to LIN desaturation). However, low-LIN flax genotypes have inactivating mutations in *FAD3a* and *FAD3b*, so *FAD3d-1*, *FAD3d-2*, *FAD3c-1*, and *FAD3c-2* were likely the major contributors to LIN synthesis in these varieties. These *FAD3* genes had low but relatively stable expression levels during seed development compared to *FAD3a* and *FAD3b*, which may explain why growth conditions had less effect on LIN content in low-LIN genotypes. High temperature with optimal watering also led to a significant increase in OLE content in flax seeds. Thus, the fatty acid composition of linseed oil is significantly altered at high temperature, which is associated with a shift to earlier stages and narrowing of the expression peaks of key *FAD* genes in seeds. These findings should be considered when cultivating flax and producing pharmaceuticals, food, paints, and other products from its seeds.

## 1. Introduction

Throughout development, plants are exposed to various adverse environmental factors, which are categorized as biotic or abiotic stressors. A number of studies were devoted to plant responses to stresses at the molecular level. Key roles of transcription factors [1,2,3,4,5,6,7,8] and signaling [9,10,11,12,13,14] were revealed in plant adaptation to biotic and abiotic pressures. High temperature and drought are two of the most destructive abiotic stressors, which result in alterations at diverse levels, including epigenetic and transcriptional, protein and metabolic, nuclear and organelle, as well as plant architecture [15,16,17,18,19,20,21,22,23,24]. Climate change, which often results in high temperatures and drought during the growing season, can affect plant growth and development, reducing crop yields and the quality of plant-derived products [25,26,27,28]. These environmental factors can have a dramatic effect on a range of oilseed crops, including altering their lipid metabolism.

Insufficient watering and high temperature generally resulted in decreased levels of linoleic (LIO, C18:2) and linolenic (LIN, C18:3) polyunsaturated fatty acids (PUFAs), as well as increased levels of oleic (OLE, C18:1) monounsaturated fatty acid (MUFA), and the effect of temperature was revealed in a greater number of studies [29]. High temperature resulted in an increase in OLE but a decrease in LIO content in sunflower [30], a decrease in LIN in soybean [31], a decrease in unsaturated fatty acid (FA) content in safflower [32], and an increase in OLE in noug [33]. Even high night temperature resulted in an increase in the LIO:LIN ratio in oilseed rape [34] and an increase in OLE (additive contribution with solar radiation) in sunflower [35]. However, high temperature decreased the OLE content and increased the LIO content in olive [36,37,38] and quinoa [39]. At the same time, different genotypes of the same species often showed some variation in response to high temperatures. For example, low-LIN genotypes of soybean [40] and oilseed rape [41] showed less response of unsaturated FA composition to the environment. In addition, the ratio of saturated to unsaturated FAs in seed oil may have adaptive value: at high temperature, seeds with higher saturated FA content will be favored because their oil provides more energy [42], whereas seeds with higher unsaturated FA content may be preferred under cold conditions [43].

Flax (*Linum usitatissimum* L.) is an important food and industrial crop, whose varieties differed greatly in LIN content: less than 5% (low-LIN), about 30–40% (mid-LIN), and higher than 50% (high-LIN) [44]. Studies in flax showed that wet and cold summers resulted in higher LIO and LIN content, while hot and dry summers resulted in higher OLE content; however, an analysis in a sample set of both low-LIN and high-LIN flax genotypes and unusual climatic conditions may lead to difficulties in statistical analysis and some unexpected results [45,46,47,48].

A key role in the conversion of OLE to LIO and LIO to LIN is played by the genes encoding fatty acid desaturases 2 (FAD2) and fatty acid desaturases 3 (FAD3), respectively [49,50]. In flax, fifteen genes of the *FAD2* and six genes of the *FAD3/7/8* families are known, and there are a number of data on the expression of these genes in different organs and tissues of flax plants [51,52,53,54,55,56,57,58,59]. *FAD2a-1*, *FAD2a-2*, and *FAD2b-2* probably play the major role in the desaturation of OLE to LIO, while *FAD3a* and *FAD3b* are the key genes in the desaturation of LIO to LIN in flax seeds [51,54,55,56,60]. It is also known that mutations in the *FAD3a* and *FAD3b* genes mainly determine the LIO:LIN ratio in linseed oil, and the differences between low-LIN (inactivating mutations in both *FAD3a* and *FAD3b*) and high-LIN varieties in LIN content reach more than 10-fold [45,51,60,61,62,63,64,65,66]. Several studies revealed that the maximum expression levels of genes involved in FA synthesis occur in flax seeds 10 to 30 days after flowering. However, the dates of the maximum expression varied depending on the study [51,52,53,56,58,67]. Furthermore, no comprehensive analysis was conducted to evaluate the expression of all *FAD* family genes during seed development under various growth conditions for flax genotypes with different oil characteristics, nor was the FA composition of flax seeds assessed for the same genotypes under the same conditions.

The optimal direction of linseed oil utilization (food, pharmaceuticals, paints) is mainly determined by the levels of LIN, LIO, and OLE [44,67,68]. However, environmental conditions, especially temperature, humidity, and hours of sunshine, can affect the chemical composition of flax seeds, including the content of unsaturated FAs, seed coat color, and the content of tocopherols and lignans, in addition to genotype [69]. Therefore, in order to predict possible modifications of flax product qualities, it is necessary to understand how climate change may affect linseed oil of varieties with different characteristics and what molecular mechanisms are responsible for this. Our work aimed to determine changes in LIN, LIO, and OLE content in oil of low-, mid-, and high-LIN flax genotypes under elevated temperature with reduced watering. We then compared these results with the data on the expression of *FAD2* and *FAD3* genes to understand how growth conditions influence gene expression and whether it is associated with the content of valuable unsaturated FAs in flax seeds.

## 2. Results

### 2.1. Influence of Temperature and Watering Conditions on the Fatty Acid Composition of Linseed Oil of Low-LIN, Mid-LIN, and High-LIN Varieties

We evaluated the effect of temperature/watering conditions on the content of OLE, LIO, and LIN in oil of low-LIN (AGT 981 and AGT 1535), mid-LIN (Raciol and AGT 422), and high-LIN (AGT 427, Entre-Rios, Norlin, Atalante, and Pechersky kryazh) flax genotypes. We tested the DNA of individual plants of varieties AGT 981 and AGT 1535 (which had impurities, as we showed previously [66]) with CAPS markers and used for further analysis only plants with expected mutations in the *FAD3a* and *FAD3b* genes.

We simulated elevated temperature (24 °C) with reduced watering (1 L per pot every three days) (ETRW—elevated temperature and reduced watering) conditions, which resemble natural conditions, where high temperature is often combined with drought. We used normal temperature (20 °C) with normal watering (1 L per pot every two days) (NTNW—normal temperature and normal watering) as standard conditions for comparison with ETRW. For further comparison, we modeled natural conditions that are opposite to ETRW: low temperature accompanied by rainfall—reduced temperature (16 °C) with elevated watering (1 L per pot every day) (RTEW—reduced temperature and elevated watering).

**Figure 1:**
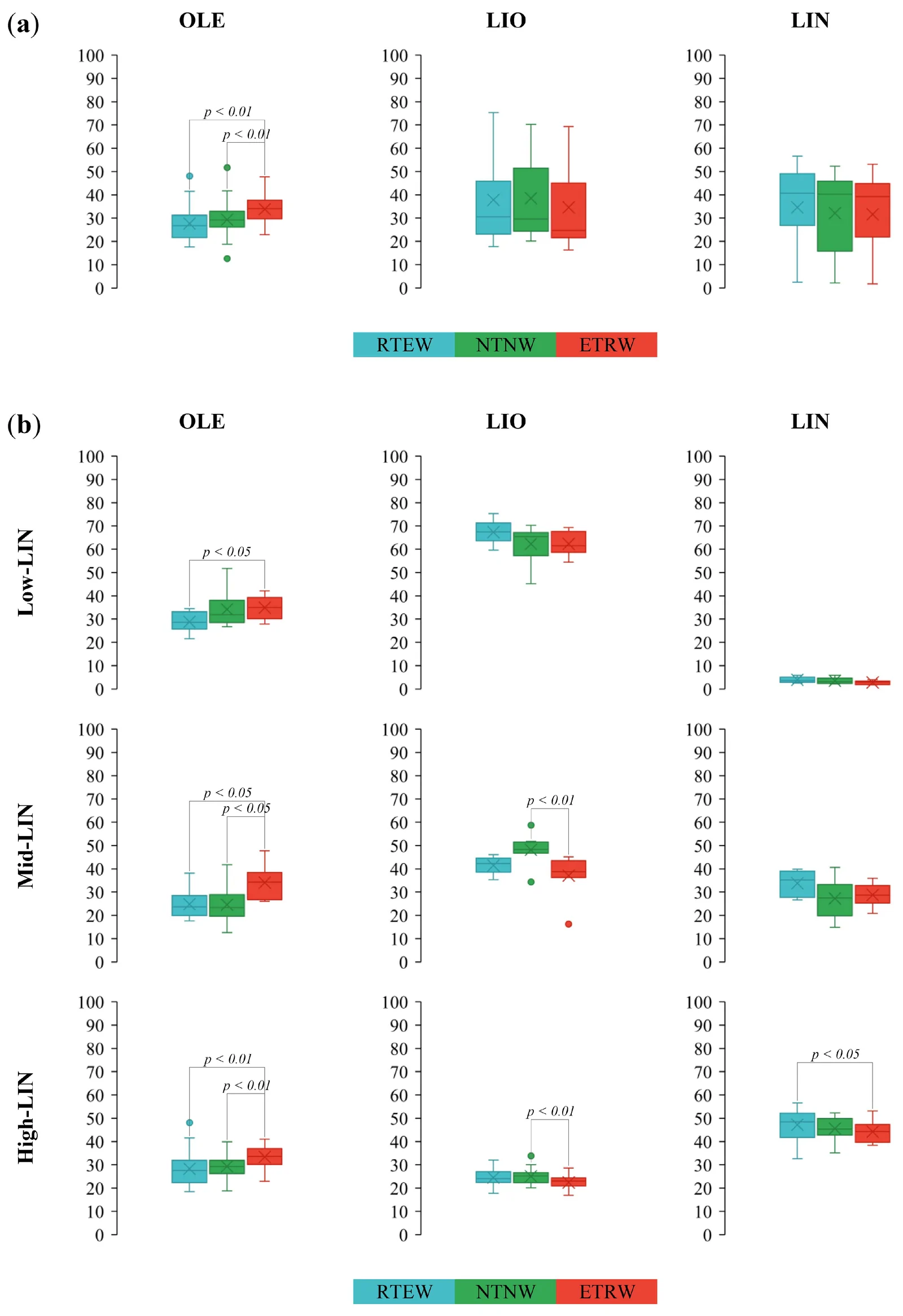
Relative percentage of unsaturated fatty acids (FAs: oleic—OLE, linoleic—LIO, and linolenic—LIN) in the oil of flax varieties under three temperature/watering conditions: reduced temperature (16 °C) and elevated watering (RTEW), normal temperature (20 °C) and normal watering (NTNW), elevated temperature (24 °C) and reduced watering (ETRW). (**a**)—Relative percentage of unsaturated FAs (OLE, LIO, and LIN) in all analyzed varieties. (**b**)—Relative percentage of unsaturated FAs (OLE, LIO, and LIN) in low-LIN, mid-LIN, and high-LIN genotypes. Rectangles correspond to the ranges containing 50% of the values (between the 25th and 75th percentiles); the horizontal line inside the rectangle is the median value (the 50th percentile); the bars (or dots in case of outliers) are the maximum and minimum values. Statistically significant differences in paired comparisons (Mann–Whitney test) are indicated with *p* < 0.01 or *p* < 0.05.

The dependence of OLE content in linseed oil on temperature/watering conditions was revealed (Figure 1a and Table S1). ETRW resulted in a significant (*p* < 0.01, Mann–Whitney test) increase in OLE content compared to NTNW (by 4.5% on average) and RTEW (by 6.2% on average). For LIO, the opposite trend was observed: ETRW resulted in an average decrease in LIO content of 4.0% compared to NTNW and 3.2% compared to RTEW. However, the differences were not statistically significant. LIN content under ETRW was lower compared to NTNW (by 0.5% on average) and RTEW (by 3.0% on average), but the differences were not statistically significant. Since we studied varieties with more than 10-fold differences in LIN content, we decided to analyze groups of genotypes with close LIN content.

Figure 1b shows the data on OLE, LIO, and LIN content for low-LIN, mid-LIN, and high-LIN flax varieties. ETRW resulted in increased OLE content compared to NTNW and RTEW in all three groups. The differences were statistically significant (*p* < 0.01 or *p* < 0.05) for mid-LIN and high-LIN genotypes. For low-LIN varieties, only the differences between the extreme conditions (ETRW vs. RTEW) were statistically significant (*p* < 0.05).

The differences in LIO content were statistically significant for mid-LIN and high-LIN varieties comparing ETRW and NTNW (*p* < 0.01), but not ETRW and RTEW, although the trend of decreasing LIO content with increasing temperature and decreasing watering was noticeable. For low-LIN genotypes, the alterations in LIO content between different temperature and watering conditions were not statistically significant.

The differences in LIN content were statistically significant only for high-LIN varieties comparing ETRW and RTEW (*p* < 0.05), but not ETRW and NTNW. No statistically significant differences in LIN content were found for mid-LIN and low-LIN genotypes depending on growth conditions.

### 2.2. Influence of Temperature and Watering Conditions on the Expression of FAD2 and FAD3 Genes in Seeds of Low-LIN, Mid-LIN, and High-LIN Flax Varieties

**Figure 2:**
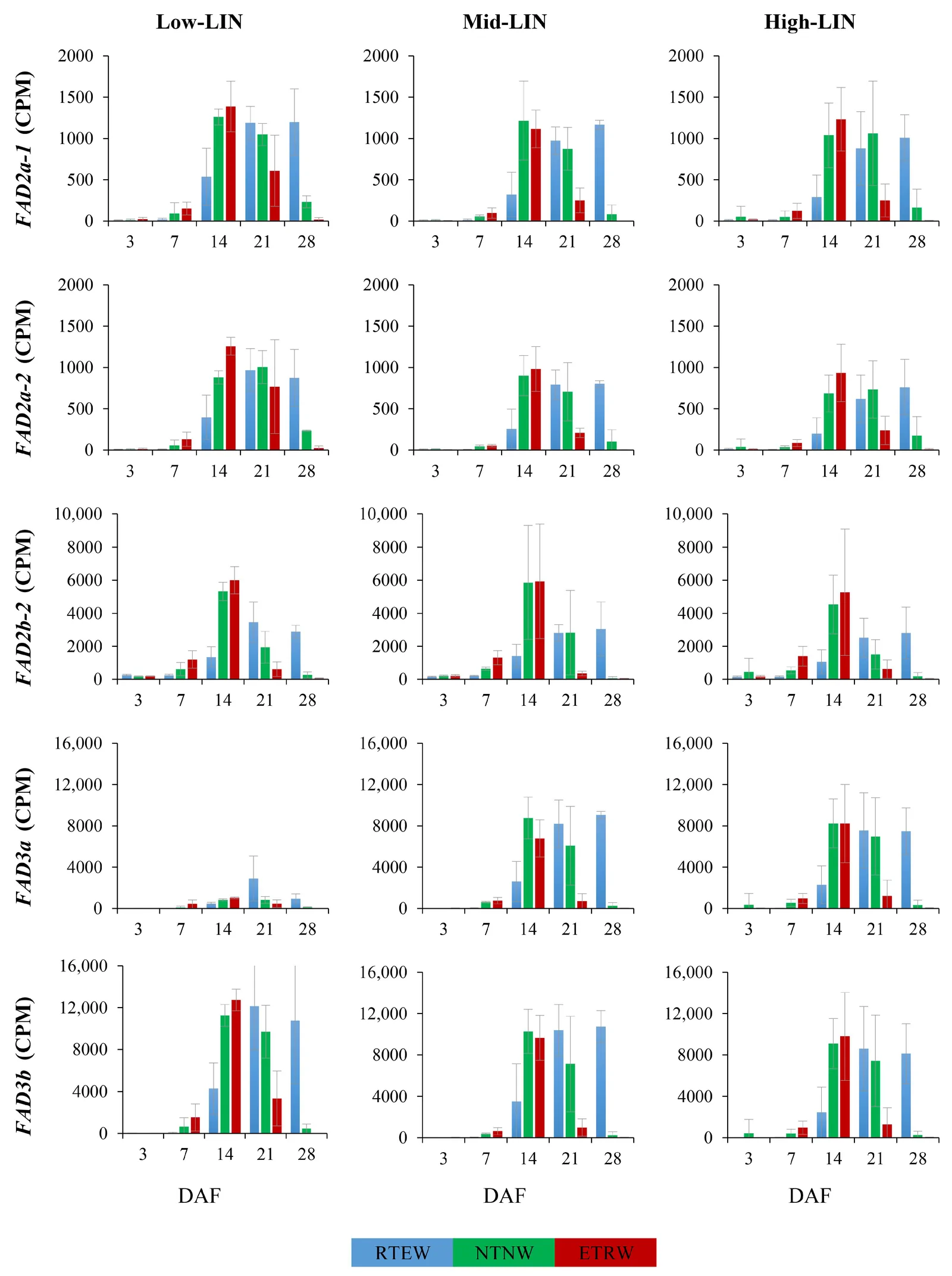
Average expression levels of *FAD2a-1*, *FAD2a-2*, *FAD2b-2*, *FAD3a*, and *FAD3b* genes (in CPM—counts per million, Y axis) in flax seeds of low-LIN, mid-LIN, and high-LIN varieties at 3, 7, 14, 21, 28 days after flowering (DAF, X axis) grown under three different temperature/watering conditions: reduced temperature (16 °C) and elevated watering (RTEW, blue), normal temperature (20 °C) and normal watering (NTNW, green), elevated temperature (24 °C) and reduced watering (ETRW, red). Error bars represent standard deviations.

We also analyzed expression changes during flax seed development under different growth conditions for *FAD2a-1*, *FAD2a-2*, *FAD2b-2*, *FAD3a*, and *FAD3b* genes, which are key for the conversion of OLE to LIO and LIO to LIN in flax seeds [54,55,56]. We used our previously obtained transcriptomic data for the same nine varieties grown under the same conditions (BioProject PRJNA1039849, NCBI Sequence Read Archive). The results are shown in Figure 2. Three groups of genotypes (low-LIN, mid-LIN, and high-LIN) showed similar trends in the expression changes in *FAD* genes. Expression levels of *FAD2* and *FAD3* increased significantly under ETRW and NTNW at 14 days after flowering (14 DAF). Under NTNW, the expression levels decreased slightly at 21 DAF and significantly at 28 DAF, while a significant expression decrease was observed already at 21 DAF under ETRW. Under RTEW, expression levels of *FAD2* and *FAD3* increased significantly at 21 DAF and remained high at 28 DAF. Thus, ETRW resulted in a shift in *FAD* gene expression profiles to earlier stages of seed development. In addition, the period of high expression levels of *FAD* genes was shorter under ETRW compared to NTNW and especially RTEW.

**Figure 3:**
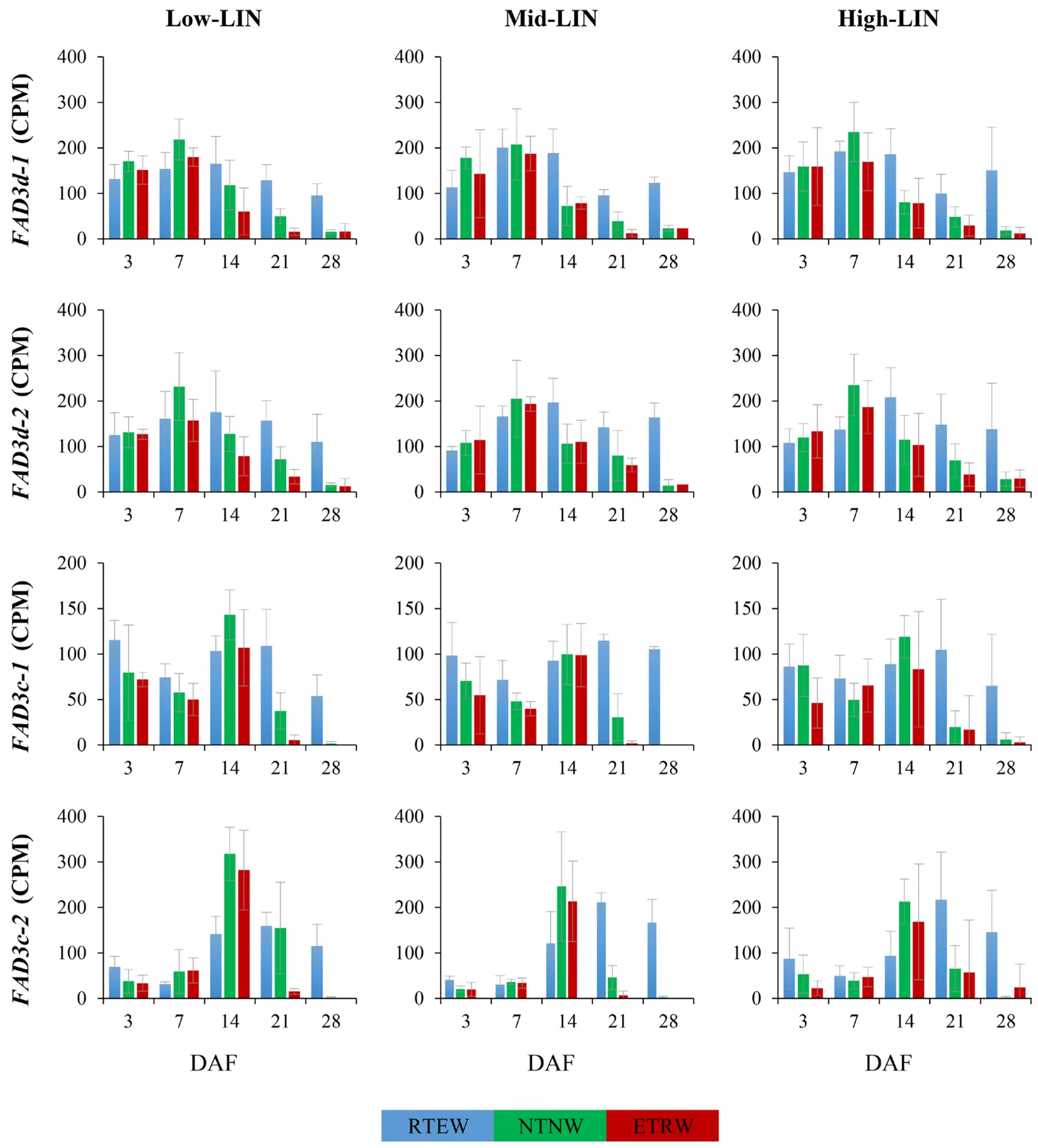
Average expression levels of *FAD3d-1*, *FAD3d-2*, *FAD3c-1*, and *FAD3c-2* genes (in CPM—counts per million, Y axis) in flax seeds of low-LIN, mid-LIN, and high-LIN varieties at 3, 7, 14, 21, 28 days after flowering (DAF, X axis) grown under three different temperature/watering conditions: reduced temperature (16 °C) and elevated watering (RTEW, blue), normal temperature (20 °C) and normal watering (NTNW, green), elevated temperature (24 °C) and reduced watering (ETRW, red). Error bars represent standard deviations.

In the present study, low-LIN genotypes showed a significantly lower level of *FAD3a* transcript with the nonsense mutation compared to varieties without the mutation, while the expression level of the *FAD3b* gene with the missense mutation was the same as in the other varieties. It should be noted that the level of LIN did not drop to zero but was maintained at 2–5% in low-LIN genotypes with inactivating mutations in both *FAD3a* and *FAD3b* genes. In addition to the *FAD3a* and *FAD3b* genes, other genes of omega-3 desaturase family were identified in flax, namely *FAD3d-1*, *FAD3d-2*, *FAD3c-1*, and *FAD3c-2* [54,55]. These genes may also be involved in the synthesis of LIN in linseed oil; therefore, we analyzed their expression (Figure 3). Expression levels of these four genes were significantly lower than the expression levels of *FAD3a* and *FAD3b* and did not differ significantly among varieties. The expression profile of *FAD3c-2* was generally similar to those of *FAD3a* and *FAD3b*. In contrast, expression levels of *FAD3d-1*, *FAD3d-2*, and *FAD3c-1* were relatively high already at early stages of seed development and were almost unaffected by growth conditions. We observed the maximum expression levels of *FAD3d-1* and *FAD3d-2* at 7 DAF and that of *FAD3c-1* at 14 DAF, but they were rather weakly pronounced. Thus, the *FAD3d-1*, *FAD3d-2*, and *FAD3c-1* genes did not show such sharp increases and decreases in expression levels during seed development, nor significant shifts in expression profiles depending on the temperature/watering conditions that were characteristic of *FAD3a* and *FAD3b*.

### 2.3. Phylogenetic Analysis of Flax Omega-3 Desaturases

The amino acid sequences of FAD3 identified in flax were compared with the amino acid sequences of omega-3 desaturases (FAD3 and FAD7/8) known for other plant species (Supplementary File S1). Six best candidates with >70% identity to AtFAD3/7/8 of *Arabidopsis thaliana* were identified in the flax variety Atlant genome (GCA_014858635.1, NCBI Genome). They corresponded to the Atlant transcripts FAD3a (H1233_027729), FAD3b (H1233_038272), FAD3c-1 (H1233_054813), FAD3c-2 (H1233_039845), FAD3d-1 (H1233_041596), and FAD3d-2 (H1233_041890) and had omega-3 FA desaturase domains and the FAD3/7/8 characteristic DUF3474 domain (pfam11960). FAD3d-1 and FAD3d-2 belonged to the FAD7/8 group, whereas FAD3a, FAD3b, FAD3c-1, and FAD3c-2 belonged to the FAD3 group (Figure S1). Taking into account that *FAD3d-1* and *FAD3d-2* had significantly different expression profiles from *FAD3a* and *FAD3b* during flax seed development, it can be speculated that FAD3d-1 and FAD3d-2 are the FAD7/8 omega-3 desaturases.

### 2.4. Influence of Temperature Conditions on the Fatty Acid Composition of Linseed Oil in F_2_ Flax Hybrids

**Figure 4:**
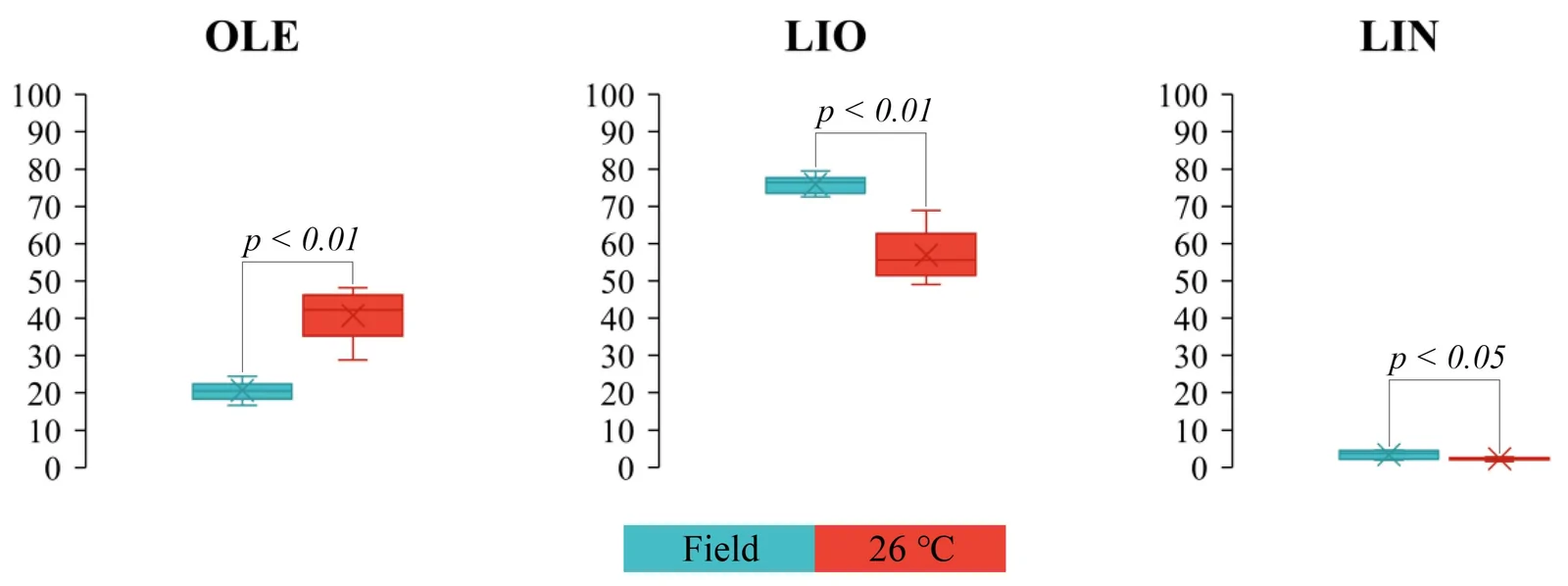
Relative percentage of unsaturated fatty acids (FAs: oleic—OLE, linoleic—LIO, and linolenic—LIN) in the oil of F_2_ flax hybrids under two different growth conditions: field with optimal watering (field) and climate chamber at 26 °C with optimal watering (26 °C). Rectangles correspond to the ranges containing 50% of the values (between the 25th and 75th percentiles); the horizontal line inside the rectangle is the median value (the 50th percentile); the bars are the maximum and minimum values. Statistically significant differences in paired comparisons (Mann–Whitney test) are indicated with *p* < 0.01 or *p* < 0.05.

To determine the effect of temperature only on the FA composition of linseed oil, we compared the content of OLE, LIO, and LIN for plants of low-LIN F_2_ flax hybrids (Lola × AGT 981/05) grown at high temperature (26 °C) and optimal watering and those grown under field conditions with an average daily temperature during flax seed ripening of about 18 °C (Figure 4 and Table S2). Statistically significant differences were found in the content of OLE (*p* < 0.01), LIO (*p* < 0.01), and LIN (*p* < 0.05) between flax plants under different growth conditions. At 26 °C, the OLE content was on average 20% higher (40.7% vs. 20.6%), the LIO content was 19% lower (56.9% vs. 75.9%), and the LIN content was 1% lower (2.3% vs. 3.4%). The experiment with the flax hybrids allowed us to evaluate the content of unsaturated FAs in seeds of plants with similar FA composition but diverse genetic bases. Furthermore, the comparison of natural field conditions with optimal watering and high temperature in a climate chamber with optimal watering revealed clear differences in OLE and LIO content in linseed oil depending on the growth conditions.

## 3. Discussion

Elevated temperature with reduced watering generally led to an increase in OLE and a decrease in LIO and LIN in linseed oil, but the changes were genotype-dependent. The greatest effect was on OLE content, and the trend was more pronounced for mid-LIN and high-LIN varieties. The decrease in LIO content was statistically significant only for mid-LIN and high-LIN genotypes. The decrease in LIN content was statistically significant only for the high-LIN group. Previous studies also revealed the influence of environmental conditions on the FA composition of linseed oil; OLE content was more affected by the environment than LIO and LIN [45,47,70,71,72,73,74,75]. However, most of the above-mentioned investigations used only wild-type high-LIN varieties or encountered difficulties in analyzing mutant low-LIN genotypes, and the experiments were performed under field conditions where it is not possible to create a specific environment. We conducted our experiment for high-LIN, mid-LIN, and low-LIN flax varieties under controlled environmental conditions, which allowed us to obtain more consistent data and minimize the weather-associated variability during the ripening of different genotypes.

To determine the causes of the changes in OLE, LIO, and LIN content in high-LIN, mid-LIN, and low-LIN groups, we analyzed the expression of *FAD* genes, which play a key role in the desaturation of OLE to LIO and LIO to LIN [51,56,60,76,77]. The expression profiles changed in a similar way for low-LIN, mid-LIN, and high-LIN groups of varieties. ETRW resulted in a shift in the maximum expression levels of these genes to earlier stages of seed development compared to NTNW and especially RTEW, as well as a shortening of the time interval with high expression levels of these genes. Previously, we described in detail the dynamics of expression levels of *FAD* genes during flax seed development [56]. The results of the present study suggest that the shift in the maximum expression levels of *FAD2a-1*, *FAD2a-2*, and *FAD2b-2* genes to earlier stages of seed development, combined with the shortening of the period of high expression of these genes, led to a significant increase in OLE content in linseed oil under ETRW. The same trend was observed for *FAD3a* and *FAD3b* and the conversion of LIO to LIN, but the differences in LIO and LIN content under different conditions were less pronounced and more genotype-dependent.

In low-LIN varieties, the differences in LIN content under different temperature/watering conditions were not statistically significant. The low-LIN genotypes had a nonsense mutation in the *FAD3a* gene and a missense mutation in the *FAD3b* gene. It was previously shown that the low-LIN mutant line of flax had reduced LIN content in seeds (up to 2%) but not in leaves [78]. Omega-3 desaturases are differentiated into microsomal (endoplasmic reticulum) (FAD3) and plastidial (chloroplast) (FAD7/8) according to their location of function [79,80,81,82,83,84]. LIN in seeds, in contrast to LIN in leaves, is mainly produced due to the activity of microsomal desaturases [85]. Vrinten et al. suggested that in low-LIN flax varieties, LIN synthesis is not due to the activity of the *FAD3a* and *FAD3b* genes carrying inactivating mutations but to the work of plastid omega-3 desaturases FAD7/8, which have low activity in seeds and are probably active at early stages of seed development [60]. We showed that genes encoding four omega-3 desaturases (*FAD3d-1*, *FAD3d-2*, *FAD3c-1*, and *FAD3c-2*) were expressed at low levels in flax seeds and may contribute to LIN synthesis from LIO. The expression of *FAD3d-1*, *FAD3d-2*, and *FAD3c-1* was much less affected by temperature/watering conditions at early stages (3, 7, and 14 DAF) than the expression of *FAD3a* and *FAD3b*. In addition, *FAD3d-1* and *FAD3d-2* had higher expression levels specifically at early stages. Amino acid sequence analysis suggested that FAD3d-1 and FAD3d-2 are plastid FAD7/8 omega-3 desaturases. Moreover, we used previously obtained transcriptomic data for flax [86] to reveal that the expression levels of *FAD3d-1*, *FAD3d-2*, *FAD3c-1*, and *FAD3c-2* were higher in leaves than in seeds, in contrast to *FAD3a* and *FAD3b*. The latter two had high expression levels in 14–21-day seeds, but low expression levels in leaves (a difference of about 10-fold). Thus, *FAD3d-1*, *FAD3d-2*, *FAD3c-1*, and *FAD3c-2* genes are likely to contribute significantly to LIN synthesis in low-LIN genotypes, and the lower dependence of *FAD3d-1*, *FAD3d-2*, and *FAD3c-1* expression profiles on temperature/watering conditions may result in less temperature/watering-dependent variation in LIN content in such genotypes. Lesser changes in FA composition under environmental conditions were also a feature of low-LIN varieties in other oilseed crops [40,41].

Analysis of literature data on changes in FA composition in seed oil of different genotypes of sunflower, oilseed rape, soybean, maize, flax, chia, safflower, olive, and camellia revealed a common trend of OLE increase and LIN and LIO decrease under high average minimum temperature during seed filling, with a more pronounced response in OLE, while OLE content in high-OLE varieties of different species had almost no response to temperature change [87]. Thus, oilseed crop genotypes with atypical FA composition may be less responsive to environmental conditions. In low-LIN flax varieties, such a lower response to temperature/watering conditions is probably due to the difference in key genes responsible for LIN synthesis compared to high-LIN genotypes.

To further evaluate the effect of temperature on FA composition of flax seeds, we analyzed the OLE, LIO, and LIN content in oil of F_2_ hybrids of two low-LIN varieties (AGT 981/05 × Lola) grown under field conditions versus conditions of high temperature (26 °C) with optimal watering. The high temperature resulted in an approximately two-fold increase in OLE content in oil and an approximately one-third decrease in LIO and LIN content, even in low-LIN genotypes, demonstrating the key role of this environmental factor in modifying the FA composition of linseed oil. Indeed, temperature is one of the key factors that affect the PUFA content in plants, usually as an inverse relationship, although other conditions, including watering, also influence this trait to some extent, especially in the field [75,88,89,90,91,92,93,94,95,96,97]. The influence of temperature on the expression of *FAD* genes and MUFA:PUFA ratio may be related to substrate availability, protein stability, isoform stability, transcriptional, post-transcriptional, and post-translational modifications [98,99].

High temperature may accelerate plant growth and development without affecting plant architecture, or it can result in thermomorphogenesis, which includes morphological and other changes [100]. High-temperature stress in oilseed crops results in faster fruit development, defective embryonic development, hollow fruits, reduced seed weight, and yield losses [101,102]. At the molecular level, numerous alterations occur in plants in response to heat stress [103]. Phytochromes [104], signaling molecules (including Ca^2+^ [105]), reactive oxygen species (ROS) [106], phytohormones [107], non-coding RNAs [108], transcription factors [109], and epigenetic regulation [110] play essential roles in the plant response to high temperature. Decreased photosynthetic activity and lipid accumulation, increased ROS, and altered membrane composition occur under heat stress [109,111,112,113,114]. Drought is also known to result in a decrease in photosynthetic pigments, increased ROS production, and cell membrane damage, which negatively affect flax flowering, seed development, and yield [115,116,117,118,119,120]. The response of plants to drought involves phytohormones, transcription factors, non-coding RNAs, and antioxidant systems [121,122,123]. Global warming results in high temperatures during plant growth, which intensifies drought. However, drought can be mitigated by watering.

**Figure 5:**
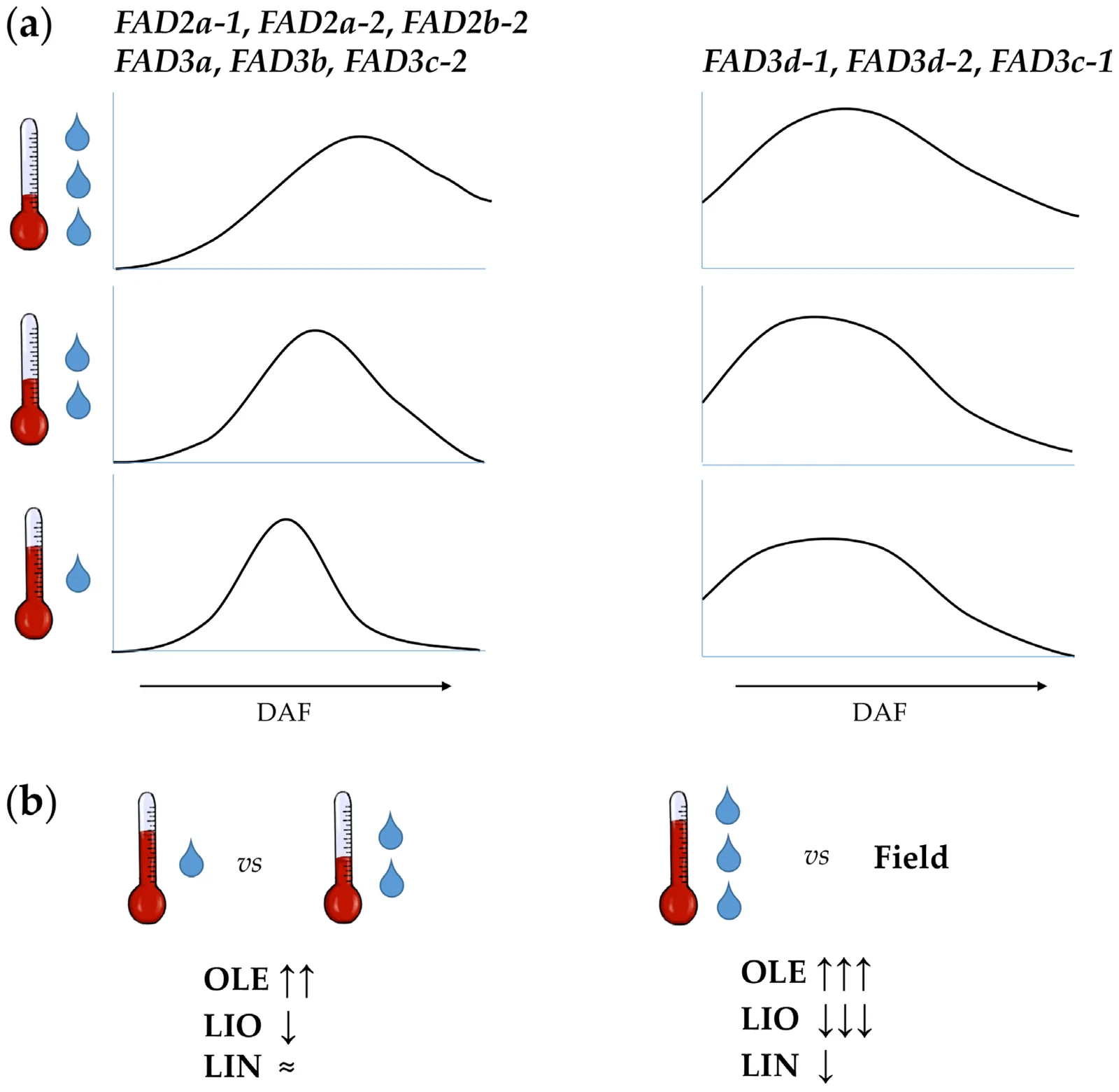
Effect of temperature and watering conditions on the expression profiles of *FAD* genes and content of unsaturated fatty acids in flax seeds. (**a**) Gene expression profiles of *FAD* genes during flax seed development under three different temperature/watering conditions: reduced temperature (16 °C) with elevated watering, normal temperature (20 °C) with normal watering, elevated temperature (24 °C) with reduced watering. DAF—days after flowering. (**b**) Alteration in the content of unsaturated fatty acids (oleic—OLE, linoleic—LIO, and linolenic—LIN) in flax seeds under elevated temperature (24 °C) with reduced watering vs. normal temperature (20 °C) with normal watering (**left panel**) and under high temperature (26 °C) with optimal watering vs. field conditions with optimal watering (**right panel**). More arrows—more significant changes.

In flax, we observed quicker ripening of capsules under high temperatures. This may result in an alteration of *FAD* gene expression profiles, predominantly a shift in the maximum expression levels to earlier stages and a shorter period of maximum expression. Certainly, other factors could be implicated in the alteration of *FAD* gene expression profiles, but their association with the rate of seed development seems quite logical. The changes in the unsaturated FA ratio that we revealed under high temperature were in good agreement with the *FAD* gene expression profiles. As the period of maximum *FAD* gene expression narrowed under high temperature, the incomplete desaturation of OLE to LIO and LIO to LIN occurred, as well as a significant increase in OLE content and a less pronounced decrease in LIO and LIN content. However, considering the similar alterations in the expression profiles of the key conversion genes of OLE to LIO (*FAD2a-1*, *FAD2a-2*, and *FAD2b-2*) and LIO to LIN (*FAD3a* and *FAD3b*) in traditional high-LIN flax varieties, it can be inferred that additional factors are implicated in the increase in OLE content in flax seeds at high temperature, and further investigation of this issue is necessary. At the same time, three of the four genes that are likely responsible for the conversion of LIO to LIN in low-LIN flax varieties (*FAD3d-1*, *FAD3d-2*, and *FAD3c-1*, with the exception of *FAD3c-2*) had different expression profiles than *FAD3a* and *FAD3b* during seed development. This could explain why low-LIN flax genotypes responded differently to high temperature than high- and mid-LIN ones did. Figure 5 illustrates the impact of growth conditions on *FAD* gene expression and the content of unsaturated FAs in flax seeds.

## 4. Materials and Methods

### 4.1. Cultivation of Flax Plants

Seeds of nine flax varieties, originating from the Czech Republic (AGT 981, AGT 1535, AGT 422, AGT 427, and Raciol), Russia (Pechersky kryazh, namely, l. 1–2 from k-2889 according to the catalog of the Federal Research Center for Bast Fiber Crops, Torzhok, Russia), Argentina (Entre-Rios), Canada (Norlin), and Netherlands (Atalante) were obtained from the Institute for Flax (Torzhok, Russia). Varieties AGT 981 and AGT 1535 had low LIN content (caused by simultaneous *FAD3a* and *FAD3b* mutations); Raciol and AGT 422 had medium LIN content (caused by the *FAD3b* mutation); AGT 427, Entre-Rios, Norlin, Atalante, and Pechersky kryazh had high LIN content (no mutations in *FAD3a* and *FAD3b*) [64,66]. Plants were grown in 15 L pots with 5 × 5 cm spacing. For each variety, 3 pots were planted with 32 seeds each. All plants were kept under the same conditions for one month and then transferred to three climate chambers with different temperature/watering conditions: 16 °C and watering 1 L per pot every day, 20 °C and watering 1 L per pot every two days, 24 °C and watering 1 L per pot every three days. We selected these conditions based on our observation that flax plants grew well and produced high-quality seeds in the climate chambers when the temperature was set to 20 °C and plants were watered every two days. We observed a decrease in seed production at temperatures above 24 °C. When the frequency of watering was reduced to less than once every three days at 24 °C, we sometimes observed a loss of turgor. At temperatures below 16 °C, the plants slowed their development, and the risk of fungal diseases increased with watering more frequently than once daily. Quantum board 301B 120-watt (Samsung lm301b 3500K + Osram Oslon 3.24 660nm + UV LG380) plant phytolamps (Minifermer, Moscow, Russia) were used to illuminate the plants with an average PPFD (photosynthetic photon flux density) = 640 μmol/m^2^/s according to the manufacturer’s recommendations.

In addition, flax plants of F_2_ hybrids obtained by crossing low-LIN varieties Lola and AGT 981/05 were studied. The crossing was performed at the Institute for Flax (Torzhok, Russia). Ten-liter pots were used to grow the parental flax lines. The plants were spaced 2.5 × 5 cm. Sowing was carried out in three rounds at one-week intervals. Crossbreeding took place from 7:00 to 10:00 a.m. during the first and second weeks of July. Maternal plants were castrated (their anthers were removed) the previous evening between 5:00 and 7:00 p.m. The flax plants were harvested at the yellow ripeness phase. The resulting F_1_ seeds were then grown in field conditions, producing F_2_ seeds, which were used in our study. F_2_ plants were grown in 2022: (1) under field conditions in Torzhok: 20 seeds in 3 replicates with 5 × 5 cm spacing, average day temperature ~22 °C (range—16–29 °C) and night temperature ~13 °C (range—8–18 °C) during general seed ripening, daily watering in dry hot weather in the morning and evening with 5 L per m^2^ and (2) in the climate chamber as described above but at 26 °C temperature and daily watering with 1 L per pot.

### 4.2. Testing Flax Varieties for Mutations in the FAD3a and FAD3b Genes

Leaves were collected from individual 4-week-old plants, frozen, and stored at −20 °C. DNA was isolated using the DNA-EXTRAN-3 kit (Syntol, Moscow, Russia). Analysis of cleaved amplified polymorphic sequences (CAPS) [124] was used to assess the conformity of plants to the variety by the presence of inactivating mutations in the *FAD3a* and *FAD3b* genes (G to A substitution in *FAD3a* at site CP027631.1:16092348 and C to T substitution in *FAD3b* at site CP027622.1:1035655 according to the flax genome assembly GCA_000224295.2/ASM22429v2, NCBI Genome) using the previously described protocol [64].

### 4.3. Mass Spectrometry Analysis of the Fatty Acid Composition of Linseed Oil

Flax plants whose seeds were used for the mass spectrometry analysis were harvested at the yellow ripeness stage. Seeds were removed from capsules and stored in paper bags at a temperature of 22–24 °C for three to four weeks until the oil was extracted. For the analysis of the FA composition of linseed oil, we adapted the methodology described by Chagovets et al. [125]. For each condition, oil extraction was performed from three seeds of each variety in four biological replicates. For F_2_ hybrids, oil was extracted from single seeds (nine seeds for each condition). One 5 mm stainless steel bead (Qiagen, Chatsworth, CA, USA) and 400 μL of cold methanol (MEOH): methyl-tert-butyl ether (MTBE) (1:3) were added to a tube with seeds and homogenized twice for 2 min at 1800 oscillations per minute using a TissueLyser II (Qiagen). Then, 400 μL of MEOH:MTBE (1:3) was added, vortexed, incubated in a thermoshaker at 4 °C for 30 min, and homogenized in an ultrasonic bath for 10 min. The sample was transferred to a 1.5 mL tube without touching the precipitate. In total, 560 μL of cold H_2_O:MEOH (3:1) was added, shaken for 1 min at 4 °C, and centrifuged for 10 min at 14,000 rpm and 4 °C. 200 μL of the upper phase was collected in two tubes and dried for 2 h in a Concentrator Plus (Eppendorf, Hamburg, Germany) at 30 °C, V-HV mode. 200 μL of MEOH: 6% KOH (4:1) was added to the tube containing the dried lipid extract, vortexed, and incubated for 2 h at 60 °C in a thermoshaker at 1400 rpm. The sample was then cooled to room temperature, 100 μL of saturated NaCl solution and 50 μL of 29% HCl were added, shaken in a thermoshaker, and centrifuged for 30 s at 12,700 rpm and 4 °C. 200 μL of chloroform:hexane (1:4) was added, shaken in a thermoshaker at 4 °C, and centrifuged for 30 s at 12,700 rpm and 4 °C. The upper phase (80 + 80 μL) was collected in a new tube, 200 μL of chloroform:hexane (1:4) was added, shaken in a thermoshaker at 4 °C, and centrifuged for 30 s at 12,700 rpm and 4 °C. The upper phase (100 + 100 μL) was collected in a new tube, 200 μL of UPLC-H_2_O was added, shaken in a thermoshaker at 4 °C, and centrifuged for 30 s at 12,700 rpm and 4 °C. The upper phase (150 + 150 μL) was collected in a new tube and dried for 40 min in a Concentrator Plus (Eppendorf) at 30 °C, V-HV mode. The dried extract was diluted in 200 μL of acetonitrile:isopropanol (7:3) and incubated for 10 min in a thermoshaker at 4 °C.

All measurements were performed on the Ultimate 3000 RSLC nano-HPLC system (Thermo Fisher Scientific, Waltham, MA, USA) coupled to the QExactive mass spectrometer (Thermo Fisher Scientific) using an ACQUITY HSS T3 column (0.075 μ  ×  10 cm, 1.8 μ, Waters Corp., Milford, MA, USA) in two replicates. The mobile phase consisted of 0.1% formic acid in LC-MS grade water (Fisher Chemical, Waltham, MA, USA, phase A) and 0.1% formic acid in acetonitrile (Fisher Chemical, phase B). Separation was held in the following gradient: 60% B at 0–2 min, increase to 85% B at 2–18 min, hold at 85% B at 18–20 min, then decrease back to 60% B at 20–22 min and re-equilibrate for 8 min. The flow rate was 0.800 μL/min, and the injection volume was 0.25 μL. Signal registration was performed in nano-ESI negative mode with a spray voltage of −1.9 kV. Resolution power was set to 35,000. The resulting full chromatograms were processed to extract the relative content of FAs. Namely, the total intensity corresponding to each FA was calculated as the sum of the signal intensities of the first and second peaks: OLE (*m*/*z* = 281.24 and 282.24), LIO (*m*/*z* = 279.23 and 280.23), and LIN (*m*/*z* = 277.21 and 278.21). The relative content of each unsaturated FA was calculated by dividing its total intensity by the sum of the total intensities of all three FAs. Then, the results were averaged over two technical replicates.

### 4.4. Expression Analysis of FAD2 and FAD3 Genes

Gene expression analysis was performed with PPline [126] as described earlier [86]. Previously obtained by us transcriptomic data (BioProject PRJNA1039849, NCBI Sequence Read Archive) were used for expression analysis of *FAD* genes as described earlier [56]. Raw reads were trimmed and filtered using Trimmomatic v0.38 [127] with the following parameters: TRAILING:24 SLIDINGWINDOW:4:14 MINLEN:40, and also residual adapters were removed. The variety Atlant genome assembly (GCA_014858635.1, NCBI Genome) [128] was used as a reference for alignment of RNA-Seq reads using STAR v2.7.3a [129]. GTF file was also supplied to STAR. Read counting per gene was performed with featureCounts v2.0.0 [130]. Average counts per million (CPM) values were calculated using the edgeR 4.0.2 package [131] for R v4.3.2. Data normalization was carried out with the TMM method. Expression levels were evaluated for the *FAD2a-1* (Atlant transcript H1233_058938), *FAD2a-2* (H1233_061927), *FAD2b-2* (H1233_078572), *FAD3a* (H1233_027729), *FAD3b* (H1233_038272), *FAD3c-1* (H1233_054813), *FAD3c-2* (H1233_039845), *FAD3d-1* (H1233_041596), and *FAD3d-2* (H1233_041890) genes for groups of low-LIN, mid-LIN, and high-LIN flax varieties.

### 4.5. Statistical Analysis

For the RNA-Seq analysis, each cDNA library was prepared from an RNA pool of five plant samples collected from the same variety under the same growth conditions and at the same stage of development to level out variations in plant material. cDNA libraries were prepared in two biological replicates. To assess expression levels of *FAD* genes in the analyzed flax samples, we used a CPM table generated by PPline. We calculated average expression levels and standard deviations and created diagrams based on these data in Excel 2016 (Microsoft, Redmond, WA, USA). For the assessment of mass spectrometry data (three seeds of each genotype in four biological replicates for varieties and one seed in nine biological replicates for F_2_ hybrids), median value (the 50th percentile), 25th and 75th percentiles, and the maximum and minimum values of relative percentage of unsaturated FAs (OLE, LIO, and LIN) in linseed oil were calculated and visualized as box plots using Excel 2016 (Microsoft). The Mann–Whitney test [132] was used to determine statistically significant differences in OLE, LIO, and LIN content within groups of flax genotypes under different growth conditions.

### 4.6. Phylogenetic Analysis of Flax Omega-3 Desaturases

*FAD3/7/8* genes in the variety Atlant genome assembly (GCA_014858635.1, NCBI Genome) were searched by protein homology with AtFAD3/7/8 sequences of *Arabidopsis thaliana* (https://www.arabidopsis.org/, accessed on 15 September 2025) using blastp with default parameters. The conserved domains of the received candidates were analyzed using the NCBI CD-batch search tool [133].

FAD3/7/8 sequences of the following species were selected for phylogenetic analysis with the flax FAD3/7/8 sequences: *Juglans regia* [134], *Populus euphratica* [135], *Arabidopsis thaliana* (https://www.arabidopsis.org/, accessed on 15 September 2025), *Camelina sativa* [136], *Perilla frutescens* [137], and *Olea europaea* [138] (See Supplementary File S1 for sequences). Multiple alignment was performed using Muscle. We inferred the maximum-likelihood tree using the IQ-TREE web server [139,140]. Branch supports were obtained using the ultrafast bootstrap [141] with 1000 replicates.

## 5. Conclusions

Growing flax plants under conditions of high temperature caused by climate change may result in seeds with increased OLE content and decreased LIO and LIN content. This trend will be particularly pronounced in high-LIN varieties, which are mainly used for the production of pharmaceutical products, paints, and varnishes [44,67]. This may have a negative impact on these products. In contrast, for low-LIN genotypes, which are used in the food industry due to their increased resistance to oxidation [44,67,68], increased OLE content may have a positive effect, further improving the stability of linseed oil. In addition, high temperature, even with optimal watering, also resulted in a significant increase in OLE and a decrease in LIO and LIN, indicating that it would not be possible to compensate for high temperature with abundant watering.

## Data Availability

The original contributions presented in this study are included in the article/Supplementary Material. Further inquiries can be directed to the corresponding author.

## Supplementary Materials

The following supporting information can be downloaded at: https://www.mdpi.com/article/10.3390/ijms27188006/s1.
