# Supplementary material for: Effect of High Temperature and Drought on the Fatty Acid Composition of Oil of Different Linseed Varieties and Its Association with Gene Expression
Source: Int J Mol Sci. 2026 Sep 9;27(18):8006. doi: 10.3390/ijms27188006 (PMC13607755; doi:10.3390/ijms27188006)
Supplement: Supplementary file 1 [file ijms-27-08006-s001.zip › Figure S1_2026.07.26.pdf]

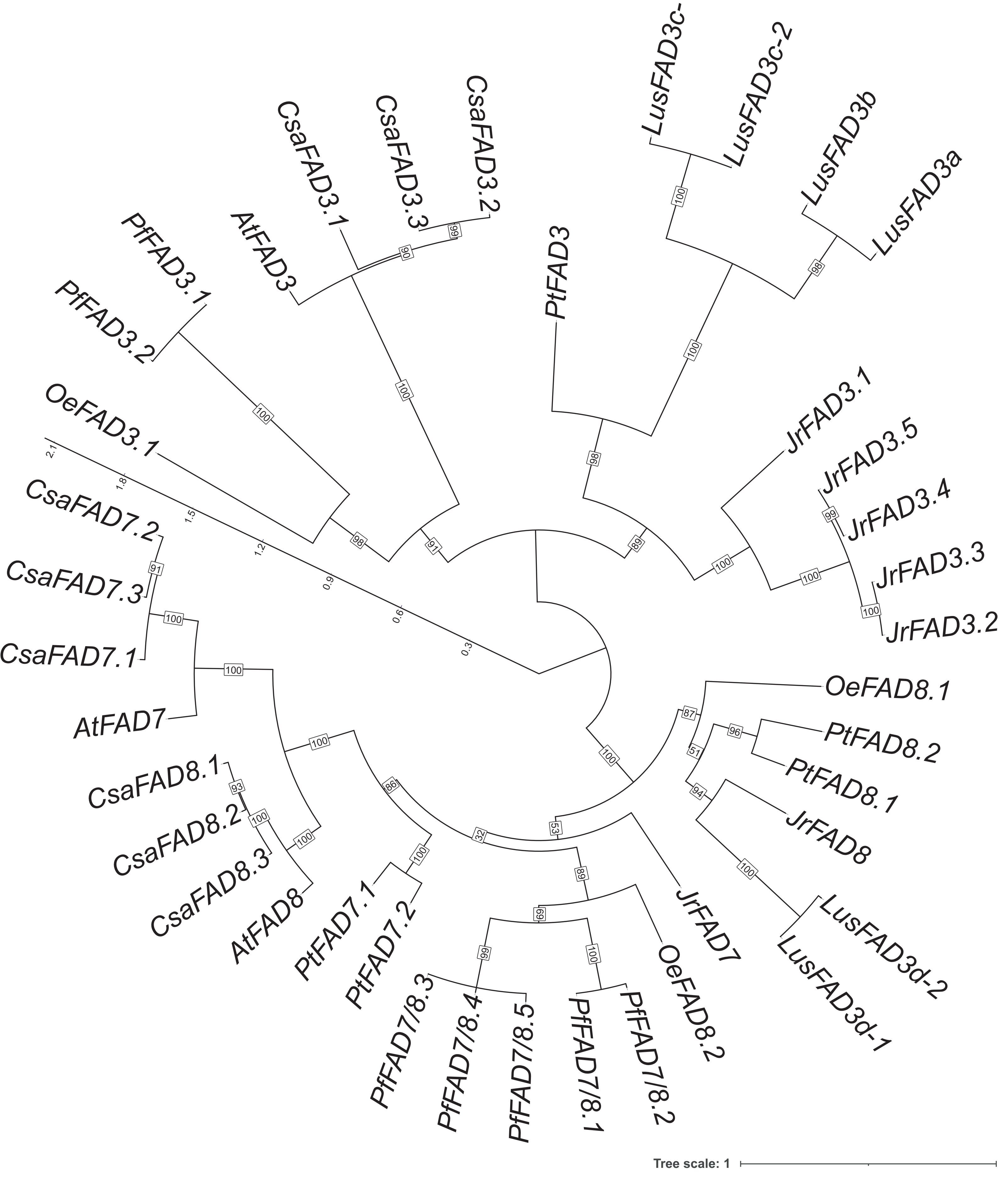

Supplementary Figure S1. Phylogenetic analysis of amino acid sequences of FAD3/7/8 of *Linum usitatissimum* (*LUSxxx*), *Juglans regia* (*Jrxxx*), *Populus euphratica* (*Ptxxx*), *Arabidopsis thaliana* (*Atxxx*), *Camelina sativa* (*Csaxxx*), *Perilla frutescens* (*Pfxxx*), *Olea europaea* (*Oexxx*). Maximum likelihood method. Bootstrap with 1000 replicates.
