# Supplementary material for: Effect of High Temperature and Drought on the Fatty Acid Composition of Oil of Different Linseed Varieties and Its Association with Gene Expression
Source: Int J Mol Sci. 2026 Sep 9;27(18):8006. doi: 10.3390/ijms27188006 (PMC13607755; doi:10.3390/ijms27188006)
Supplement: Supplementary file 1 [file ijms-27-08006-s001.zip › File S1_2026.07.26.pdf]

Supplementary File S1. Amino acid sequences of FAD3/7/8 used for phylogenetic analysis: *Linum usitatissimum* (*LUSxxx*), *Juglans regia* (*Jrxxx*), *Populus euphratica* (*Ptxxx*), *Arabidopsis thaliana* (*Atxxx*), *Camelina sativa* (*Csaxxx*), *Perilla frutescens* (*Pfxxx*), *Olea europaea* (*Oexxx*).

>*JrFAD3.1*

MEKGGENANGFGHGIGHKDDDFDPSAPPPFRIAEIRAAIPKHCWIKNPWRSLSYVLRDFF
MVFALAAAAIYLNTWIFWPLYWAAQGTMFWAIFVLGHDCGHGSFSNSTLLNSFMGHL
LHSAILVPYNGWRISHRTHHQNHGNVENDESWVPLTEKLYKSLDQSTRKLRFTVPFPLF
AYPFYLWSRSPGKEGSHFNPYSNLFAPNERKDVITSTLCWSLMAALLIYLSFAVGPIQML
KLYGVPHLIFVMWLDFVTYLHHHGYEQKLPWYRGKEWSYLRGGLTTVDRDYGIFNNI
HHDIGTHVIHHLFPQIPHYHLVEATKAAKPVLGKYYREPKKSGPIPLHLIESLVRSITEDH
YVDDSGEIVYYQTDPELFKSLKSKAI

>*JrFAD3.2*

MEGGGGEKNASVFSEFGPTDVFDPSAPPPFKIAEIRAAIPKHCWIKSSWRSLSYVLRDLL
VIFVLAAAAIYFNAGIFWPFYWVAQGTMFWAIFVLGHDWRISHRTHHQNHGNVENDES
WVPLTEKLYKSLDYSTKMLRFTVPFPLLAYPFYLWTRGPGKEGSHFNPNSKLFAPNDRN
DVITSTVCWSIMVALLISLSFAIGPIQMLKLYCVPYLIFVMWLDFVTYLQHHGYEQKLPW
YRGKEWSYLRGGLTTVDRDYGWINNIHHDVGTHLIHHLFPQIPHYHLVEATEAAKPVL
GKYYREPKKSGPIPFHLIQNLARSIKEDHYVSDSGEIVYYQTDSELYKSSRDKSN

>*JrFAD3.3*

MEGGGGEKNASVFSEFGPTDVFDPSAPPPFKIAEIRAAIPKHCWIKSSWRSLSYVLRDLL
VIFVLAAAAIYFNAGIFWPFYWVAQGTMFWAIFVLGHDCGHGSFSDNSVLNSLVGHVL
HSAILVPYHGWRISHRTHHQNHGNVENDESWVPLTEKLYKSLDYSTKMLRFTVPFPLLA
YPFYLWTRGPGKEGSHFNPNSKLFAPNDRNDVITSTVCWSIMVALLISLSFAIGPIQMLK
LYCVPYLIFVMWLDFVTYLQHHGYEQKLPWYRGKEWSYLRGGLTTVDRDYGWINNIH
HDVGTHLIHHLFPQIPHYHLVEATEAAKPVLGKYYREPKKSGPIPFHLIQNLARSIKEDH
YVSDSGEIVYYQTDSELYKSSRDKSN

>*JrFAD3.4*

MKEPVLEEMEGGGGEKNASVFSGFGQTDVFDPSAPPPFKIAEIRAAIPKHCWIKSSWRSL
SYVLRDLLVIFALAAAAIYFNAGIFWPFYWVAQGTMFWAIFVLGHDWRISHRTHHQNH
GNVENDESWVPLTEKLYKSLDYSTKMLRFTVPFPLLAYPFYLWTRGPGKEGSHFNPNSK
LFAPNDRNDVITSTVCWSIMVALLISLSFAIGPIQMLKLYCVPHLIFVMWLDFVTYLHHH
GYEQKLPWYRGKEWSYLRGGLTTVDRDYGWINNIHHDIGTHVIHHLFPQIPHYHLVEA
TEAAKPVLGKYYREPKKSGPIPFHLIQNLARSIKEDHYVSDSGEIVYYQTDSEFYKSSKY
KSN

>*JrFAD3.5*

MKEPVLEEMEGGGGEKNASVFSGFGQTDVFDPSAPPPFKIAEIRAAIPKHCWIKSSWRSL
SYVLRDLLVIFALAAAAIYFNAGIFWPFYWVAQGTMFWAIFVLGHDCGHGSFSDNSVL
NSLVGHVMHSAILVPYHGWRISHRTHHQNHGNVENDESWVPLTEKLYKSLDYSTKML
RFTVPFPLLAYPFYLWTRGPGKEGSHFNPNSKLFAPNDRNDVITSTVCWSIMVALLISLSF
AIGPIQMLKLYCVPHLIFVMWLDFVTYLHHHGYEQKLPWYRGKEWSYLRGGLTTVDR
DYGWINNIHHDIGTHVIHHLFPQIPHYHLVEATEAAKPVLGKYYREPKKSGPIPFHLIQNL
ARSIKEDHYVSDSGEIVYYQTDSEFYKSSKYKSN

>*JrFAD7*

MASWVLTECGLKPLPRIFPRLRHGLASNSSFPSEVRPLPIFKSYRLVSSLGVSSGFRGNSW
ALNVSAPLRVTSVGEGDENRERDSGVNGLEDEGEFDPGSPPPFRLADIRAAIPKHCWVK
DPWRSMSYVVRDVVVVFGLAAVAAYFNHWAVWPLYWIAQGTMFWALFVLGHDCGH
GSFSNNPNLNNVVGHLLHSSILVPYHGWRISHRTHHQNHGHVENDESWHPLSEKIYKNL
DNVTRTLRFTFPFPMLAYPFYLWGRSPGKKGSHFHPNSDLFVPNERKDVITSTACWTAM
AALLGYLSFAMGPLQMLKLYFIPYWIFVMWLDLVTYLHHHGHEEKLPWYRGKEWSYL
RGGLTTLDRDYGIINNIHHDIGTHVIHHLFPQIPHYHLVEATEAAKPVLRKYRREPKKSGP
LPFHLVGTLIRSMKQDHYVSETGDVVYYQTDPQLYGPEASKASK

>*JrFAD8*

MASWVISECGLKPLPQIFPRPTTGPVSCKPSKIRFLHSNKTGTDLKFPSTKILTGCFRERN
WGLKVSAPLRVASVEGDEEREQRIDGVNVLKEEEGQPEFDPSAPPPFKLADIRAAIPKHC
WVKDPWRSMSYVARDVAVVFGLAATAAYFNNWFVWPLYWAAQGTMFWALFVLGH
DCGHGSFSNNPKLNSVVGHLLHSSILVPYHGWRISHRTHHQNHGHVENDESWHPLSEKI
FKNLDNITRTLRFTVPFPLLAYPFYLWNRSPGKTGSHFDPKSDLFVPTERKDVITSTVCW
TAMAALLGVLAFVMGPVQLLKLYGVPYGGFVMWLDLVTYLHHHGHEDKLPWYRGKE
WSYLRGGLTTIDRDYGWINNIHHDIGTHVIHHLFPQIPHYHLIEATEAAKPVLGKYYREP
KKSGPLPFYLIGSLIKSLRQDHYVSDSGDVVYYKTDPKLSGSSSK

>*PtFAD3*

MKQSCLERSNKKDASNDTVDGIDGEGDFDPSAPPPFKIAEIRAAIPKHCWVKNPWRSLS
YVLRDVTVVFALAAAVLRLDSWVFWPLYWAAQGTMFWAIFVLGHDCGHGSFSNNHL
LNNVVGHVLHSSILVPYHGWRISHRTHHQNHGNVENDESWVPLPEKIYKNLDLPTRIMR
YTVPLPMLAYPIYLWRRSPGKEGSHFNPYSKLFAHSERKAVLTSTVCWSIMVLLLIYSSY
VFDPVQVLKIHGVPYLIFVMWLDFVTYLHHHGHEQKLPWYRGKEWSYLRGGLTTVDR
DYGWINGIHHDIGTHVIHHLFPQIPHYHLVEATRAAKPVLGKYYREPKKSGPFPLHLFSN
LVRSIREDHYVSDTGDVVYYQTDPQLCTIKSN

>*PtFAD7.1*

MASWVLSECGLRPLPRIYPQSRTGLTSKSTNLLKLRQSPDSKSCNLGSPFKVSSCSRQRN
WALNVAVPVSVPVCEEEDKERESVNGANEQEGGFFDPGAPPPFKLSDIRAAIPKHCWVK
NPWISMSYVVRDVAVVFGLAAIAAYFNNWVVWPLYWFAQGTMFWALFVLGHDCGHG
SFSNNPKLNSVVGHLLHSSILVPYHGWRISHRTHHQNHGHVENDESWHPLPEKIYRSLD
NVTRKLRFTLPFPMLAYPVYLWSRSPGKKGSHFHPDSDLFVPNERKDVITSTACWTAM
AALLVSLSFVMGPFQVLKLYGIPYWIFVMWLDLVTYLHHHGHDEKLPWYRGKEWSYL
RGGLTTLDRDYGWINNIHHDIGTHVIHHLFPQIPHYHLVEATEAAKPVLGKYYREPRKSE
PLPFYLLGTLVRSMKQDHYVSNTGDVLYYQTDPELCGPEKTE

>*PtFAD7.2*

MASWVLSECGLRPLPRVYHQSRTGLTSKTTNLLKIRQPPVSKSYNLGSSFKVSSWSRQR
NWALNVAIPVSAPVIEEEDKERESVKGVNEHEGEFFDPGAPPPFKLADIRAAIPKHCWVK
NPWRSMSYVVRDVAVVCGLAVVAAYFNNWVVWPLYWFAQGTMFWALFVLGHDCG
HGSFSNNPKLNSVVGHLLHSSILVPYHGWRISHRTHHQNHGHVENDESWQPLPEKIYKS
LDNVTRTLRFTLPFPMLAYPVYLWSRSPGKKGSHFHPDSDLFAPNERKDVITSTACWTA
MAALVACLSFVMGPVQMLKLYGIPYWIFVMWLDLVTYLHHHGHDEKLPWYRGKEWS
YLRGGLTTLDRDYGWINNIHHDIGTHVIHHLFPQIPHYHLIEATEAAKPVLGKYYREPKK
SGPLPFYLLGTLIRSMKQDHYVSDAGDVLYYQTDPKLYGPEKTE

>*PtFAD8.1*

MASWVLSECGLKPLPQFWPRPRTGIINPSKPRFLYTNRTLTDLKRLPFDKFSRGCFRERN
WGLKVSAPYRVASLDGEEDNKVIGFNGVGEDEETEFDPGSPPPFKLSDIRTAIPKHCWV
KDPWKSMSYVVRDVAVVFGLAAAAAYFNNWVVWPLYWAAQGTMFWALFVLGHDC
GHGSFSNNNKLNSVAGHILHSSILVPYHGWRISHRTHHQNHGHVENDESWHPLPEKIYK
SLDGITKMLRFTVPFPMLAYPIYLWSRSPGKSGSHFDPNSDLFVPSERKDVITSTLCWTA
MAALLVGLSFVMGPVQLLKLYGVPYVGFVMWLDLVTYLHHHGHEDKLPWYRGKEWS
YLRGGLTTIDRDYGWINNIHHDIGTHVIHHLFPQIPHYHLIEATEAARPVFGKYYREPKKS
GPLPFHLIGILIRSMRKDHYVSDTGDVVYYQTDPQHGGSSSE

>*PtFAD8.2*

MASLVLSECGLKPLPQTLHRPRTGIISHNNPSKTRFLFTNRTLTDIKRLPFDKFSRGCFRER
NWGLKVSAPFKVAYGEEDNKINGFNGLGEEEIEFDPGSPPPFNLSDVRAAIPKHCWVKD
PWRSMSYVVRDVAVVFGLAAVAAYLNNWLVWPLYWAAQSTMFWAIFVLGHDCGHG
SFSNNHNLNSVVGHILHSSILVPYHGWRISHRTHHQNHGHVEKDESWHPLSEKIYKSLD
DMTKKLRFTVPFPMLAYPIYLWSRSPGKTGSHFHPNSDLFVPSEKIDVITSTVCWTAMV
ALLVGLSFVMGPIQLLNLYGIPYVGFVMWLDLVTYLHHHGHEDKLPWYRGKEWSYLR
GGLTTLDRDYGWINNIHHDIGTHIIHHLFPQIPHYHLIEATEAAKPVLGKYYREPKKSGPL
PFHLIGDLIRSMARDHYVSDTGDVVYYQTDSQVSRSSSEE

>*LusFAD3d-1*

MASWVLSECGIKPLPSAFPKPRTGALSRNTLPKLRYLPPRSNLSAAADGGASDLSLNLPK
LSSVEKKKPLLGQVRVTAPFKVAPVKEEDGEGESNFDPGAPPPFNLADIRAAIPKHCWV
KDPWRSMAYVARDVAVVLGLAAAAAYLNNWIVWPLYWAAQGTMFWALFVLGHDC
GHGSFSSNHKLNSVVGHILHSSILVPYHGWRISHRTHHQNHGHVENDESWHPLSEKIFRS
LDATTRSMRFTLPFPMLAYPFYLWNRSPGKKGSHFDPSSDLFVASEKKDVITSTVCWTA
MAALLAGLSFVMGPIQMIKLYGIPYWGFVMWLDFVTYLHHHGHDDKLPWYRGKEWS
YLRGGLTTIDRDYGWINNIHHDIGTHVIHHLFPQIPHYHLIEATEAAKPVLGKYYKEPAK
SKPLPFHLIGDLIKSLKRDHYVSDTGDVVYYQTDPELQRPSSSS

>*LusFAD3d-2*

MASWVLSECGIKPLPSAFPKPRTGALSRNTLPKLRYLPPRSNLSAAADGGASDLSLNLPK
LSSVEKKRPLLGQVRVTAPFKVAPVKEEDGGGESNFDPGAPPPFNLADIRAAIPKHCWV
KDPWRSMAYVARDVAVVLGLAAAAAFLNNWIVWPLYWAAQGTMFWALFVLGHDCG
HGSFSSNHKLNSVVGHILHSSILVPYHGWRISHRTHHQNHGHVENDESWHPLSEKIFRSL
DATTRSMRFTLPFPMLAYPFYLWNRSPGKKGSHFDPSSDLFVASEKKDVITSTVCWTAM
AALLAGLSFVMGPIQMIKLYGIPYWGFVMWLDFVTYLHHHGHDDKLPWYRGKEWNY
LRGGLTTIDRDYGWINNIHHDIGTHVIHHLFPQIPHYHLIEATEAAKPVLGKYYKEPAKS
KPLPFHLIGDLIKSLKRDHYVSDTGDVVYYQTDPELQRPSSSS

>*LusFAD3a*

MSDAMETETSTVTEPSSKMIHDYSDFKTVALHDQTMSPPNSMSPATNGSTNGVAINGA
KKLLDFDPSAAPPFKIADIRAAIPPHCWVKNPWRSLSYVLRDLLVILSFAVAATKLDSWT
VWPLYWIAQGTMFWAVFVLGHDCGHGSFSDSWLLNNVMGHILHSSILVPYHGWRISH
KTHHQNHGNVEKDESWVPLPEKVYKSLDTGTKFMRFTIPLPMFAYPIYLWRRSPGKKG
SHFNPYSDLFAPNERTSVMISTLCWTAMALLLCYSSFIYGFLPVFKIYGVPYLIFVAWLD
MVTYLHHHGYEQKLPWYRGKEWSYLRGGLTTVDRDYGVINNIHHDIGTHVIHHLFPQ

MPHYHLVEATQAAKHVLGKYYREPKKSGPFPFHLFGYLVRSLGEDHYVSDTGDVVFY
QSDPHIPKFPTSATTKSKSS

>*LusFAD3b*

MSDAMETETSTVTEPSSKMIHDYSDFKTVALQDQTMSPPNSMSPTTNGNGVAMNGAK
KQLDFDPSAAPPFKIADIRAAIPPHCWVKNPWRSLSYVLRDLLVILSFAVAAAKLDSWTF
WPLYWVAQGTMFWAVFVLGHDCGHGSFSDIWLLNNVMGHILHSSILVPYHGWRISHK
THHQNHGNVEKDESWVPLPEKVYKSLDTSTKFMRFTIPLPMFAYPIYLWTRSPGKKGSH
FNPYSDLFAPNERAAVLISTLCWTAMALLLCYSSFIYGFAPVLKIYGVPYLIFVAWLDMV
TYLHHHGYEQKLPWYRGKEWSYLRGGLTTVDRDYGVINNIHHDIGTHVIHHLFPQMPH
YHLVEATQAAKHVLGKYYREPKKSGPFPFHLFGYLVRSLGEDHYVSDTGDVVFYQSDP
HIPKFRTSSATTKSKSS

>*LusFAD3c-2*

MSPPNSMGMEAAHPTGNGNGVAVMNGSAANKPDFDPSEAPPFKIADIRAAIPPHCWVK
NPWRSLSYVLRDAVVILAFAAAALKLDLWAVWPLYWIAQGTMFWAVFVLGHDCGHG
SFSDSWLLNNVVGHILHSAILVPYHGWRISHKTHHQNHGNVEKDESWVPLPEKVYKTL
DTSTKFMRFTIPLPMFAYPIYLWTRSPGKKGSHFNPYSDLFAPQERKSVLISTISWISMVLI
LLYASFLFGFLTVFKVYTVPYLIFVAWLDMVTYLHHHGHEEKLPWYRGKEWSYLRGGL
TTVDRDYGIINNIHHDIGTHVIHHLFPQIPHYHLVEATKAAKSVLGKYYREPKKSGPFPFH
LFDNLVRSLGEDHYVSDAGDVVFYQSDPDIFKFSKSKSA

>*LusFAD3c-1*

MSPPNSMGMEAAHPTGNGNGVAVMNGASANKPDFDPSEAPPFKIADIRAAIPPHCWVK
NPWRSLSYVLRDAVVILAFAAAALKLDLWAVWPLYWIAQGTMFWAVFVLGHDCGHG
SFSDSWWLNNVVGHILHSAILVPYHGWRISHKTHHQNHGNVEKDESWVPLPEKVYKTL
DTSTKFMRFTIPLPMFAYPIYLWTRSPGKKGSHFNPYSDLFAPQERKSVLISTISWISMVLI
LLYASFLFGFLTVFKVYTVPYLIFVAWLDMVTYLHHHGHEEKLPWYRGQEWSYLRGGL
TTVDRDYGIINNIHHDIGTHVIHHLFPQIPHYHLVEATKAAKSVLGKYYREPKKSGPFPFH
LFDNLVRSLGEDHYVSDAGDVVFYQSDPEIFKFSKSKSA

>*AtFAD3*

MVVAMDQRTNVNGDPGAGDRKKEERFDPSAQPPFKIGDIRAAIPKHCWVKSPLRSMSY
VVRDIIAVAALAIAAVYVDSWFLWPLYWAAQGTLFWAIFVLGHDCGHGSFSDIPLLNSV
VGHILHSFILVPYHGWRISHRTHHQNHGHVENDESWVPLPERVYKKLPHSTRMLRYTVP
LPMLAYPLYLCYRSPGKEGSHFNPYSSLFAPSERKLIATSTTCWSIMFVSLIALSFVFGPL
AVLKVYGVPYIIFVMWLDAVTYLHHHGHDEKLPWYRGKEWSYLRGGLTTIDRDYGIFN
NIHHDIGTHVIHHLFPQIPHYHLVDATKAAKHVLGRYYREPKTSGAIPIHLVESLVASIKK
DHYVSDTGDIVFYETDPDLYVYASDKSKIN

>*AtFAD7*

MANLVLSECGIRPLPRIYTTPRSNFLSNNNKFRPSLSSSSYKTSSSPLSFGLNSRDGFTRN
WALNVSTPLTTPIFEESPLEEDNKQRFDPGAPPPFNLADIRAAIPKHCWVKNPWKSLSYV
VRDVAIVFALAAGAAYLNNWIVWPLYWLAQGTMFWALFVLGHDCGHGSFSNDPKLNS
VVGHLLHSSILVPYHGWRISHRTHHQNHGHVENDESWHPMSEKIYNTLDKPTRFFRFTL
PLVMLAYPFYLWARSPGKKGSHYHPDSDLFLPKERKDVLTSTACWTAMAALLVCLNFT
IGPIQMLKLYGIPYWINVMWLDFVTYLHHHGHEDKLPWYRGKEWSYLRGGLTTLDRD

YGLINNIHHDIGTHVIHHLFPQIPHYHLVEATEAAKPVLGKYYREPDKSGPLPLHLLEILA
KSIKEDHYVSDEGEVVYYKADPNLYGEVKVRAD

>*AtFAD8*

MASSVLSECGFRPLPRFYPKHTTSFASNPKPTFKFNPPLKPPSSLLNSRYGFYSKTRNWAL
NVATPLTTLQSPSEEDTERFDPGAPPPFNLADIRAAIPKHCWVKNPWMSMSYVVRDVAI
VFGLAAVAAYFNNWLLWPLYWFAQGTMFWALFVLGHDCGHGSFSNDPRLNSVAGHL
LHSSILVPYHGWRISHRTHHQNHGHVENDESWHPLPESIYKNLEKTTQMFRFTLPFPML
AYPFYLWNRSPGKQGSHYHPDSDLFLPKEKKDVLTSTACWTAMAALLVCLNFVMGPIQ
MLKLYGIPYWIFVMWLDFVTYLHHHGHEDKLPWYRGKEWSYLRGGLTTLDRDYGWI
NNIHHDIGTHVIHHLFPQIPHYHLVEATEAAKPVLGKYYREPKNSGPLPLHLLGSLIKSM
KQDHFVSDTGDVVYYEADPKLNGQRT

>*CsaFAD3.1*

LHISFLPLYKQITQTPKNSSNPFFFSPHFSSFTEPILRSSLFLSLSLSLSPEMVVAMDKRSNV
NGDPGAGDRNKKGEGFDPSAQPPFKIGDIRAAIPKHCWVKSPLRSMSYVVRDICAVAAL
AVAAVYFNSWFLWPLYWAAQGTLYWAIFVLGHDCGHGSFSDIPLLNSVVGHILHSFILV
PYHGWRISHRTHHQNHGHVENDESWVPLPERVYKNLPHSTRMLRYTVPLPMLAYPLYL
CYRSPGKEGSHFNPYSSLFAPSERKLIATSTTCWSIMFVTLIALSFIFGPLSVLKVYGVPYII
FVMWLDAVTYLHHHGHDEKLPWYRGKEWSYLRGGLTTIDRDYGIFNNIHHDIGTHVIH
HLFPQIPHYHLVDATKSAKHVLGRYYREPQTSGAIPIHLVESLVASIKKDHYVSDTGDIV
FYETDPDLYVYASDKSKIN

>*CsaFAD3.2*

MVVAMDKRSNVNGDPGAGDRNKKGEGFDPSAQPPFKIGDIRAAIPKHCWVKSPLRSMS
YVVRDICAVAALAVAAVYFNSWFLWPLYWAAQGTLFWAIFVLGHDCGHGSFSDIPLLN
SVVGHILHSFILVPYHGWRISHRTHHQNHGHVENDESWVPLPERVYKNLPHSTRMLRYT
VPLPMLAYPLYLCYRSPGKEGSHFNPYSSLFAPSERKLIATSTTCWSIMFVTLIALSFIFGP
LSVLKVYGVPYIIFVMWLDAVTYLHHHGHDEKLPWYRGKEWSYLRGGLTTIDRDYGIF
NNIHHDIGTHVIHHLFPQIPHYHLVDATKAAKHVLGRYYREPQTSGAIPIHLVESLVASIK
KDHYVSDTGDIVFYETDPDLYVYASDKSKIN

>*CsaFAD3.3*

MVVAMDKRSNVNGDPGAGDQKKKGEGFDPSAQPPFKIGDIRAAIPKHCWVKSPLRSMS
YVVRDICAVAALAVAAVYFNSWFLWPLYWAAQGTLFWAIFVLGHDCGHGSFSDIPLLN
SVVGHILHSFILVPYHGWRISHRTHHQNHGHVENDESWVPLPERVYKNLPHSTRMLRYT
VPLPMLAYPLYLCYRSPGKEGSHFNPYSSLFAPSERKLIATSTTCWSIMFVTLIALSFIFGP
LSVLKVYGVPYIIFVMWLDAVTYLHHHGHDEKLPWYRGKEWSYLRGGLTTIDRDYGIF
NNIHHDIGTHVIHHLFPQIPHYHLVDATKAAKHVLGRYYREPQTSGAIPIHLVESLVASIK
KDHYVSDTGDIVFYETDPDLYVYASDKSKIN

>*CsaFAD7.1*

MANLVLSECGIRPLPRIYTTPRSNFVSNNNKPIFKFRPFTSYKTSSSPLACSRDGFGKNWS
LNVSVPLTTTTPIVDESPLKEEEEEKQRFDPGAPPPFNLADIRAAIPKHCWVKNPWKSMS
YVLRDVAIVFALAAGAAYLNNWIVWPLYWLAQGTMFWALFVLGHDCGHGSFSNNPRL
NNVVGHLLHSSILVPYHGWRISHRTHHQNHGHVENDESWHPMSEKIYQSLDKPTRFFRF
TLPLVMLAYPFYLWARSPGKKGSHYHPESDLFLPKEKTDVLTSTACWTAMAALLICLNF
VVGPVQMLKLYGIPYWINVMWLDFVTYLHHHGHEDKLPWYRGKEWSYLRGGLTTLD

RDYGVINNIHHDIGTHVIHHLFPQIPHYHLVEATEAVKPVLGKYYREPDKSGPLPLHLLGI
LAKSIKEDHYVSDEGDVVYYKADPNMYGEIKVGAD

>*CsaFAD7.2*

MANLVLSECGIRPLPRIYTTPRSNFISNNNKPIFKFRPLTSYKTSSPLACSRDGFGGRNWSL
NVSVPLTTTTPIVDESPLEEEEEEKQRFGPGAPPPFNLADIRAAIPKHCWVKSPWKSMSY
VLRDVAIVFALAAGAAYLNNWIVWPLYWLAQGTMFWALFVLGHDCGHGSFSNDPRLN
NVVGHLLHSSILVPYHGWRISHRTHHQNHGHVENDESWHPMSEKIYQSLDKPTRFFRFT
LPLVMLAYPFYLWARSPGKKGSHYHPESDLFLPKEKTDVLTSTACWTAMAALLICLNF
VVGPVQMLKLYGIPYWINVMWLDFVTYLHHHGHEDKLPWYRGKEWSYLRGGLTTLD
RDYGVINNIHHDIGTHVIHHLFPQIPHYHLVEATEAVKPVLGKYYREPDKSGPLPLHLLGI
LAKSIKEDHYVSDEGEVVYYKADPNMYGEIKVGAD

>*CsaFAD7.3*

MANLVLSECGIRPLPRIYTTPRSNFVSNNNKPIFKFRPLTSYKTSSPLFCSRDGFGRNWSL
NVSVPLATTTPIVDESPLEEEEEEEKQRFDPGAPPPFNLADIRAAIPKHCWVKNPWKSMS
YVLRDVAIVFALAAGAAYLNNWIVWPLYWLAQGTMFWALFVLGHDCGHGSFSNNPRL
NNVVGHLLHSSILVPYHGWRISHRTHHQNHGHVENDESWHPMSEKIYQSLDKPTRFFRF
TLPLVMLAYPFYLWARSPGKKGSHYHPESDLFLPKEKTDVLTSTACWTAMAALLICLNF
VVGPVQMLKLYGIPYWINVMWLDFVTYLHHHGHEDKLPWYRGKEWSYLRGGLTTLD
RDYGVINNIHHDIGTHVIHHLFPQIPHYHLVEATEAVKPVLGKYYREPDKSGPLPLHLLGI
LAKSIKEDHYVSDEGDVVYYKADPNMYGEIKVGAD

>*CsaFAD8.1*

MASWVLSECGIRPLPRFYPKPTNSFVSNPTPTFKFKFNPTIKPISSTLDSREGFFSKTRNWT
LNVATPLTTVQSPSEEDTERFDPGAPPPFNLADIREAIPKHCWVKNPWMSMSYVVRDVA
IVFGLAALASYFNNWLLWPLYWFAQGTMFWALFVLGHDCGHGSFSNDRRLNSVAGHL
LHSSILVPYHGWRISHRTHHQNHGHVENDESWHPLPESMYKKLEKATQIFRFTLPFPML
AYPFYLWNRSPGKNGSHYHPDSDLFLPKEKKDVLTSTACWTAMAALLVCLNFVMGPIQ
MLKLYGIPYWIFVMWLDFVTYLHHHGHEDKLPWYRGKEWSYLRGGLTTLDRDYGWI
NNIHHDIGTHVIHHLFPQIPHYHLVEATEAAKPVLGKYYREPKNSGPLPLHLLGSLIKSM
KQDHFVSDTGDVVYYETDPKLN

>*CsaFAD8.2*

MASWVLSECGIRPLPRFYPKPTNSFVSNPTPTFKSKFNPTISPLSSSLDSREGFFSKTRNWT
LNVATPLTTLQSPSEEHTERFDPGAPPPFNLADIREAIPKHCWVKNPWMSMSYVVRDVA
IVFGLAALASYFNNWLLWPLYWFAQGTMFWALFVLGHDCGHGSFSNDPRLNSVAGHL
LHSSILVPYHGWRISHRTHHQNHGHVENDESWHPLPESMYKKLEKATQIFRFTLPFPML
AYPFYLWNRSPGKNGSHYHPDSDLFLPKEKKDVLTSTACWTAMAALLVCLNFVMGPIQ
MLKLYGIPYWIFVMWLDFVTYLHHHGHEDKLPWYRGKEWSYLRGGLTTLDRDYGWI
NNIHHDIGTHVIHHLFPQIPHYHLVEATEAAKPVLGKYYREPKNSGPLPLHLLGSLIKSM
KQDHFVSDTGDVVYYEKDPKLN

>*CsaFAD8.3*

MASWVLSECGIRPLPRFYPKPTNSFVSNPTPTFKFKFNPTITPLSSSLDSRKGFFSKTRNW
TLNVATPLTTLESPSEEDTERFDPGAPPPFNLADIRAAIPKHCWVKNPWMSMSYVVRDV
AIVFGLAALASYFNNWLLWPLYWFAQGTMFWALFVLGHDCGHGSFSNDPRLNSVAGH
LLHSSILVPYHGWRISHRTHHQNHGHVENDESWHPLPESMYKKLEKATQIFRFTLPFPM

LAYPFYLWNRSPGKNGSHYHPDSDLFLPKEKKDVLTSTACWTAMAALLVCLNFVMGPI
QMLKLYGIPYWIFVMWLDFVTYLHHHGHEDKLPWYRGKEWSYLRGGLTTLDRDYGW
INNIHHDIGTHVIHHLFPQIPHYHLVEATEAAKPVLGKYYREPKNSGPLPLHLLGSLIKSM
KQDHFVSDTGDVVYYETDPKLN

>*PfFAD3.1*

MAVSSGARLSKSGADGEVFDGQQQYEGIGKRAADKFDPAAPPPFKIADIRAAIPAHCWV
KNPWRSLSYVVWDVAAVFALLAAAVYINSWAFWPVYWIAQGTMFWALFVLGHDCGH
GSFSDNTTLNNVVGHVLHSSILVPYHGWRISHRTHHQNHGHVEKDESWVPLPENLYKK
LDFSTKFLRYKIPFPMFAYPLYLWYRSPGKTGSHFNPYSDLFKPNERGLIVTSTMCWAA
MGVFLLYASTIVGPNMMFKLYGVPYLIFVMWLDTVTYLHHHGYDKKLPWYRSKEWSY
LRGGLTTVDQDYGFFNKIHHDIGTHVIHHLFPQIPHYHLVEATREAKRVLGNYYREPRK
SGPVPLHLIPALLKSLGRDHYVSDNGDIVYYQTDDELFPSKKI

>*PfFAD3.2*

MAVSSGARLSKSGADGEVFDGQQQYEGIGKRAADKFDPAAPPPFKIADIRAAIPAHCWV
KSPWRSLSYVVWDVAAVFALLAAAVYINSWAFWPVYWIAQGTMFWALFVLGHDCGH
GSFSDNTTLNNVVGHVLHSSILVPYHGWRISHRTHHQNHGHVEKDESWVPWYRSPGKT
GSHFNPYSDLFKPNERGLIVTSTMCWAAMGVFLLYASTIVGPNMMFKLYGVPYLIFVM
WLDTVTYLHHHGYDKKLPWYRSKEWSYLRGGLTTVDQDYGFFNKIHHDIGTHVIHHLF
PQIPHYHLVEASCSTSYSTREAKRVLGNYYREPRKSGPVPLHLIPALLKSLGRDHYVSDN
GDIVYYQTDDELFPSKKI

>*PfFAD7/8.1*

MASFVISECGLKPLPRIYPKPRAAQPLSSSNLRFSRTNQRFNSSFCSSSGINKERNWALRV
SAPLRIQPVEEENRAINGGEEFDPAAPPPFKLSDIKAAIPKHCWVKDPWRSVSYVVRDVV
AVFGMAAAAAYFNNWLVWPLYWFAQSTLFWALFVLGHDCGHGSFSNNPKLNSVFGH
LLHSSILVPYHGWRISHRTHHQNHGHVENDESWHPLPEKIYNSLDNNTKMLRFTLPFPM
LAYPFYLWSRSPGKKGSHFHPESDLFVPNERKDVITSTVCWTAMAALLVGLSFVIGPLQ
LLKLYGIPYLGFVAWLDLVTYLHHHGHEDKLPWYRGKEWSYLRGGLTTLDRDYGLIN
NIHHDIGTHVIHHLFPQIPHYHLIEATEAAKGVLGKYYREPKKSGPLPLHLLGELLRSMK
KDHYVSDTGDIVYYQTDPQLNGGRKS

>*PfFAD7/8.2*

MASFVISECGLKPLPRIYPKPRAAQPLSSSNLRFSRTNQRFNSSFCSSTGIIKERNWALRVS
APLRIQPVEEENRAINGGEEFDPAAPPPFKLSDIKAAIPKHCWVKDPWRSVSYVVRDVV
AVFGMAAAAAYFNNWLVWPLYWFAQSTLFWALFVLGHDCGHGSFSNNPKLNSVFGH
LLHSSILVPYHGWRISHRTHHQNHGHVENDESWHPLPEKIYNSLDNNTKMLRFTLPFPM
LAYPFYLWSRSPGKKGSHFHPESDLFVPNERKDVITSTVCWTAMAALLVGLSFVIGPLQ
LLKLYGVPYLGFVAWLDLVTYLHHHGHEDKLPWYRGKEWSYLRGGLTTLDRDYGLIN
NIHHDIGTHVIHHLFPQIPHYHLIEATEAAKGVLGKYYREPKKSGPLPLHLLGDLLRSMK
KDHYVSDTGDIVYYQTDPQLNGGRKS

>*PfFAD7/8.3*

MASWVLSECGLRPLPRIYPKPRTDQYVSNSNPSRLRVSRTGFSSDSSFSLVGRERNWGLK
VSAPLRFQEVEEESEERGSVVVNGVDEFDPGAPPPFKLSDIRAAIPKHCWVKDPWRSMS
YVVRDVVVVFGLAAGAAYFNNWAVWPLYWFAQSTMFWALFVLGHDCGHGSFSNDP
KLNSVAGHLLHSSILVPYHGWRISHRTHHQNHGHVENDESWHPIPEKIYKTLDFATKKL

RFTLPFPMLAYPFYLWGRSPGKKGSHFHPDSDLFVPNERKDVITSTVCWTAMLAILAGL
SFVMGPVQLLKLYGIPYIGFVAWLDLVTYLHHHGHDEKLPWYRGKEWSYLRGGLTTL
DRDYGWINNIHHDIGTHVIHHLFPQIPHYHLIEATAAAKPVLGKYYKEPKKSGPFPFYLL
GVLQKSMKKDHYVSDTGDIVYYQTDPELN

>*PfFAD7/8.4*

MAASLQLNCKPFTPLLLHSRSASKRAPKFSTVRCSAASPSKSYTITLLPGDGIGPEVISVA
KNALKLVASLEGFAFKFTEVPMGGAALDLTGVPLPEETLSIAKGSDAVLLGAIGGYKWD
NNEKHLKPETGLLQLRAGLKVFANLRPATVLPQLVDASTLKKDVAEGVDLMVVRELTG
GIYFGKPRGFGKNENGEEIGFNTEVYTTHEIDRIARVAFETARKRGGKLCSVDKANVLE
ASMLWRKRVTALASEYPDVELSHMYVDNAAMQLVRNPKQFDTIVTNNIFGDILSDEAS
MITGSIGMLPSASLGESGPGLFEPIHGSAPDIAGQDKANPLATVLSAAMLLKYGLGEEKA
ARRIEAAVLDTLDRGYRTGDIHSAGQKLVGCREMGEQVLKSIDSKVATAVGHGSFSND
PKLNSVAGHLLHSSILAPYHGWRISHRTHNQNHGHVENDESWHPIPEKIYRTLDFATKK
LRFTLPFPMLAYPFYLLGRSPGKKGSHFHPDSDLFVPIERKDVITSTVCWTAMVAGLSFV
TGPAHLSEFHASLYNLKMELGLQGFVAWLDLVTYLHHHGHDEKLPWYRGMELPETGA
HDT

>*PfFAD7/8.5*

MAASLQLNCKPFTPLLLHSKSASKRAPKFSTVHCSAASPSKSYTITLLPGDGIGPEVISVA
KNALKLVASLEGFAFKFTEVPVGGAALDLTGVPLPEETLSIAKGSDAVLLGAIGGYKWD
NNEKHLKPETGLLQLRAGLKVFANLRPATVLPQLVDASTLKKDVAEGVDLMVVRELTG
GIYFGKPRGFGKNENGEEIGFNTEVYTTHEIDRIARVAFETARKRGGKLCSVDKANVLE
ASMLWRKRVTALASEYPDVELSHMYVDNAAMQLVRNPKQFDTIVTNNIFGDILSDEAS
MITGSIGMLPSASLGESGPGLFEPIHGSAPDIAGQDKANPLATVLSAAMLLKYGLGEEKA
ARRIEAAVLDTLDRGYRTGDIHSAGQSGEEMGEQVLKSIDSKVTIAVGHGSFSNDPKLN
SVAGHLLHSSILVPYHGWRISHRTHHQNHGHVENDESWHPIPEKIYRTLDFATKKLRFTL
PFPMLAYPFYLWGRSPGKKGSHFHPDSDLFVPNERKDVITSTVCWTAMVAGLSFVTGP
AQLLKTLWHTLYWICGMA

>*OeFAD3.1*

MAIGLSHSYSLPSADKNNEDELMKKVCFSPLENGNEVVNGDEIDFDPSEPPPFKIGDIRA
AIPEHCWVKDPWRSCSYVVRDIAVALGLVGMALHFNSWIHWPVYWFAQGTIFWAIFVL
GHDCGHGSFSDNPNLNNIVGHILHSSILVPYHGWRISHRTHHQNHGHVENDESWVPLPE
KIYKKLDMSTKFLRYKVPFPLFAYPLYLWSRSPGKKGSHFNPYSDLFKSSERKLVLTSTV
CWAAMVAFLFCLSFRFGPLLLLKLYGVPYVIFVMWLDLVTYLHHHGHEKKLPWYRGK
EWSYLRGGLTTVDRDYGWFNKIHHDIGTHVIHHLFPQIPHYHLEEATSAAKQVMGMYY
REPIKSGPVPYHLLQNLVRSVKEDHFVSDSGDIVYYQKDAQLS

>*OeFAD8.1*

MAGWVLSECGIKPLPQIFPKPRTSFFSNSIKNHTHLQKVQFLHANKKILNPKFSPGSIPRLS
RDKIWVLNVSAPSRIASLDEYEKGEKMETIDGVNGIGEEEGEFDCGAPPPFKLADIRAAIP
KHCWVKDPWRSMSYVVRDVAIVFGLAAGAAYLNNWFIWPLYWAAQGTMFWAFFVL
GHDCGHGSFSNNQKLNSVVGHILHSSILVPYHGWRISHRTHHQNHGHVENDESWHPLP
EKIYKNLDKVTQTLRFTVPFPMLAYPFYLWNRSPGKTGSHFDPNSDLFIPNERKDVITSTI
CWTAMAALLAGLSFVLGPVKLLQLYGVPYWGFVMWLDFVTYLHHHGHEDKLPWYRG
KEWSYLRGGLTTLDRDYGWINNIHHDIGTHVIHHLFPQIPHYHLVEATEAAKPVLGEYY
REPKKSSPLPFHLIGDLVKSLEQDHYVSDTGDVVYYQTDPNLSSSNKSK

>OeFAD8.2

MASWVLSECGLKPLPRFYAKPRTGQALLNSNPSKVRISRTSGNGYSFSLSSGFRERNWG
LKVSAPLRVQPVEENKEEGKILNGDESFDPGAPPPFKLSDIKAAIPKHCWVKDPWKSMS
YVVRDVVVVFGLAAVAAYFNNWVFWPLYWFAQGTMFWALFVLGHDCGHGSFSNNP
KLNSVAGHLLHSSILVPYHGWRISHRTHHQNHGHVENDESWHPLSEKIYKNLDNVTKK
LRFTLPFPLLAYPIYLWSRSPGKTGSHYHPDSDLFVPNERKDVITSTVCWTAMVAILAGL
SFIMGPVQLLKLYGIPYAIFVMWLDLVTYLHHHGHDAKLPWYRGKEWSYLRGGLTTLD
RDYGWVNNIHHDIGTHVIHHLYPQIPHYHLVEATEAAKPVLGKYYREPKKSGPLPLYLL
GVLIRSMRKDHYVSDSGDIVYYQTDSQL
